# Disruption of miRNA-mRNA Networks Defines Novel Molecular Signatures for Penile Carcinogenesis

**DOI:** 10.3390/cancers13194745

**Published:** 2021-09-23

**Authors:** Tatiane Katsue Furuya, Claudio Bovolenta Murta, Alexis Germán Murillo Carrasco, Miyuki Uno, Laura Sichero, Luisa Lina Villa, Leonardo Cardilli, Rafael Ferreira Coelho, Giuliano Betoni Guglielmetti, Mauricio Dener Cordeiro, Katia Ramos Moreira Leite, William Carlos Nahas, Roger Chammas, José Pontes

**Affiliations:** 1 Center for Translational Research in Oncology (LIM24), Instituto do Cancer do Estado de Sao Paulo (ICESP), Hospital das Clinicas da Faculdade de Medicina da Universidade de Sao Paulo (HCFMUSP), Sao Paulo CEP 01246-000, SP, Brazil; agmurilloc@usp.br (A.G.M.C.); miyuki.uno@hc.fm.usp.br (M.U.); laura.sichero@hc.fm.usp.br (L.S.); l.villa@hc.fm.usp.br (L.L.V.); rchammas@usp.br (R.C.); 2 Departamento de Urologia, ICESP, HCFMUSP, Sao Paulo CEP 01246-000, SP, Brazil; rafael.coelho@hc.fm.usp.br (R.F.C.); giuliano.betoni@hc.fm.usp.br (G.B.G.); mauricio.cordeiro@hc.fm.usp.br (M.D.C.); katiaramos@usp.br (K.R.M.L.); wnahas@uol.com.br (W.C.N.); docjpjr@uol.com.br (J.P.J.); 3 Departamento de Patologia, ICESP, HCFMUSP, Sao Paulo CEP 01246-000, SP, Brazil; leonardo.cardili@hc.fm.usp.br

**Keywords:** penile cancer, miRNA, gene expression levels, carcinogenesis, integrative analysis

## Abstract

**Simple Summary:**

As there are still no biomarkers reported in clinical practice in penile cancer (PeC), we aimed to investigate and validate molecular signatures based on miRNA and mRNA profiles to identify molecular drivers and pathways involved in PeC tumorigenesis. We found eight DEmiRs and 37 DEGs comparing tumoral tissues (TT) paired with non-neoplastic tissues (NNT) of PeC patients. Four downregulated DEmiRs (miR-30a-5p, miR-432-5p, miR-487b-3p, and miR-145-5p) and six upregulated DEGs (*IL1A*, *MCM2*, *MMP1*, *MMP12*, *SFN* and *VEGFA*) were identified as potential biomarkers in PeC by their capacity of discriminating TT and NNT with accuracy. Furthermore, we performed an analysis of miRNA-mRNA interaction and found disruption in the dynamics of the regulation of eight pairs during tumor development that have never been described in PeC. Taken together, our findings contribute to a better understanding of the regulatory roles of miRNAs and altered transcripts levels in penile carcinogenesis.

**Abstract:**

Penile cancer (PeC) carcinogenesis is not fully understood, and no biomarkers are reported in clinical practice. We aimed to investigate molecular signatures based on miRNA and mRNA and perform an integrative analysis to identify molecular drivers and pathways for PeC development. Affymetrix miRNA microarray was used to identify differentially expressed miRNAs (DEmiRs) comparing 11 tumoral tissues (TT) paired with non-neoplastic tissues (NNT) with further validation in an independent cohort (*n* = 13). We also investigated the mRNA expression of 83 genes in the total sample. Experimentally validated targets of DEmiRs, miRNA-mRNA networks, and enriched pathways were evaluated in silico. Eight out of 69 DEmiRs identified by microarray analysis were validated by qRT-PCR (miR-145-5p, miR-432-5p, miR-487b-3p, miR-30a-5p, miR-200a-5p, miR-224-5p, miR-31-3p and miR-31-5p). Furthermore, 37 differentially expressed genes (DEGs) were identified when comparing TT and NNT. We identified four downregulated DEmiRs (miR-30a-5p, miR-432-5p, miR-487b-3p, and miR-145-5p) and six upregulated DEGs (*IL1A*, *MCM2*, *MMP1*, *MMP12*, *SFN* and *VEGFA*) as potential biomarkers in PeC by their capacity of discriminating TT and NNT with accuracy. The integration analysis showed eight dysregulated miRNA-mRNA pairs in penile carcinogenesis. Taken together, our findings contribute to a better understanding of the regulatory roles of miRNAs and altered transcripts levels in penile carcinogenesis.

## 1. Introduction

Penile cancer (PeC) is a rare neoplasm with high incidence in low and middle-income countries [1]. It was estimated about 36,000 new cases worldwide in 2020 [2], and Brazil stands out among the countries with the highest incidence rates globally, mainly in the north and northeast region [1,3]. Its treatment relies mainly on mutilating surgeries even in the early stages of the disease, with more than 60% of patients undergoing partial or total penectomy [4]. Moreover, almost half of the patients are still submitted to bilateral inguinal lymphadenectomy which is accompanied by complications in two thirds of the cases [5,6]. Some risk factors have been described in PeC etiology, including poor penile hygiene, low socioeconomic status, phimosis, chronic inflammation, tobacco use, immunosuppression, and human papillomavirus (HPV) infection [7].

Genes and molecular pathways altered in penile carcinogenesis include: (i) the expression of high-risk HPV E6 and E7 genes, whose products interfere respectively with P53 and pRb; (ii) MAPK/ERK pathway and (iii) PI3K-AKT-mTOR axis [7,8]. In summary, penile carcinogenesis is driven by both HPV-dependent and HPV-independent pathways [7]. Recently, epigenetic factors have gained attention. DNA methylation profiles have been associated with deregulated transcript expression driving PeC development and progression [9].

To our knowledge, to date, only 11 studies have investigated the differential expression of microRNAs (miRNAs) in this disease [10,11,12,13,14,15,16,17,18,19,20]. miRNAs are a class of small non-coding RNAs (ncRNAs) that regulate gene expression post-transcriptionally by repressing translation and/or initiating mRNA degradation [21]. These small regulatory RNAs are predicted to target and silence several different mRNAs simultaneously [22]. In 2015, Zhang et al. published the first study regarding miRNA expression profile in PeC [12]. Kuasne et al. (2017) have contributed to a more comprehensive approach describing differentially expressed miRNAs and transcripts capable of distinguishing tumors from non-neoplastic tissues with high sensitivity and specificity [14].

The present study defined and validated potential molecular signatures based on miRNA and gene expression profiles in penile carcinogenesis, comparing pairs of tumoral tissues and matched adjacent non-neoplastic tissues obtained from PeC patients. Moreover, we performed an integrative analysis of miRNA and mRNA expression profiles looking for molecular drivers and pathways involved in tumor development with clinical relevance.

## 2. Materials and Methods

### 2.1. Patients and Sample Collection

From July 2015 to January 2018, patients with newly diagnosed and localized or locally advanced pathologically confirmed squamous cell carcinoma (SCC) of the penis with an indication for surgical treatment with curative intent were prospectively enrolled in our study. All PeC patients were negative for HIV, Hepatitis B, or C infections. Fresh frozen PeC primary tumoral tissues (TT) and matched adjacent non-neoplastic tissues (NNT) were collected in liquid nitrogen and stored at −80 °C for posterior nucleic acid extraction in our Academic Biobank for Research on Cancer at the University of Sao Paulo (USP), Instituto do Cancer do Estado de São Paulo (ICESP), São Paulo, Brazil. The NNT tissues were small specimens of apparently normal skin adjacent to the tumor that were removed by the surgeon and the absence of tumor was confirmed microscopically by our pathologists. The Biobank was approved by local and National Ethics Committees (Protocols 031/2012 and 023/2014, respectively).

Medical history and physical examination data were obtained during hospital admission. Patients were subjected to standardized questionnaires that included lifestyle information and socio-demographic indicators. Pathological tumor data, such as tumor size, histology, grade, staging, lymphovascular and perineural invasions, margins, and lymph node metastasis, were collected from the patients’ anatomopathological reports and medical records. Tissue slices were stained with hematoxylin and eosin and analyzed by one pathologist of our institution. Tumors were staged following the 2016 Union for International Cancer Control (UICC) TNM classification for PeC [23].

The disease was staged by computed tomography (CT) imaging of the chest, abdomen, and pelvis. Patients underwent partial or total penectomy according to the extent of the disease and were prospectively followed from the time of enrollment until April 2021 accordingly with the European Association of Urology (EAU) Guidelines on Penile Cancer [24]. Patients with T1 disease and well or moderately differentiated tumor and no signs of inguinal metastases (either by physical examination or CT scan) were only followed up, whereas those presenting clinical metastases or T1 disease with poorly differentiated tumor or ≥T2 disease were submitted to bilateral inguinal lymphadenectomy.

This study was approved by the Local Ethics Committee of the Faculdade de Medicina da Universidade de Sao Paulo (Protocol Code 1.016.980) and carried out under the terms of the Helsinki Declaration. The written informed consent and epidemiological questionnaire were obtained from all participants enrolled in the study.

### 2.2. Total RNA Extraction

Total RNA was extracted from 30 mg of PeC fresh frozen tissue fragments using miRVana^®^ (Thermo Fisher Scientific, Waltham, MA, USA), according to the manufacturer’s instructions, after quality control of those tissues. RNA concentration and purity were determined using the NanoDrop ND1000 (Thermo Fisher Scientific), and the 2200 TapeStation Instrument (Agilent Technologies, Santa Clara, CA, USA) was used to evaluate RNA integrity. Only samples with A260/A280 ratio between 1.8 and 2.1 and RNA Integrity Number (RIN) above seven were included in the microarray analysis.

### 2.3. miRNA Expression Profiling by Microarray Analysis

Samples from 11 PeC patients were used for miRNAs expression profiling using the GeneChip miRNA 4.0 Array (Thermo Fisher Scientific) following the manufacturer’s protocol. Each sample was hybridized on the array, washed, stained with the Affymetrix Fluidics Station 450, and scanned using Affymetrix GeneChip Scanner 3000 7G. Expression Console software (Thermo Fisher Scientific) was used for quality assessment. Transcriptome Analysis Console (TAC) software version 4.0.2 (Thermo Fisher Scientific) was used for background correction, normalization, and summarization of raw data (CEL files) by Robust Multichip Average plus Detection Above Background (RMA + DABG), considering probesets for mature Homo sapiens miRNAs. We used the Linear Models for Microarrays (LIMMA) test with at least two-fold changes (FC > 2), *p* < 0.01, and false discovery rate (FDR) < 0.05 to identify DEmiRs between TT (*n* = 11) and NNT (*n* = 11) groups. Hierarchical clustering graphs with the DEmiRs selected for further validation were also constructed using the TAC software. Microarray datasets are available on the Gene Expression Omnibus (GEO) database (accession number GSE172095).

### 2.4. Validation of DEmiRs by Quantitative Reverse Transcription Polymerase Chain Reaction (qRT-PCR)

Nine DEmiRs identified from microarray analysis were selected for further validation by qRT-PCR in an independent set of PeC samples (*n* = 13). The criteria for DEmiRs selection were based on *p*-value, FC, biological relevance, and a careful review of the literature searching for miRNAs deregulated or implicated in PeC or other type of SCC.

Initially, total RNA was reverse transcribed to cDNA using TaqMan^®^ Advanced miRNA cDNA Synthesis Kit (Thermo Fisher Scientific). miRNA levels were quantified using TaqMan^®^ methodology (Thermo Fisher Scientific) in the StepOnePlus Real-Time PCR system (Thermo Fisher Scientific), using the default cycling conditions recommended by the manufacturer. The Taqman^®^ Advanced miRNA Assays (Thermo Fisher Scientific) of the selected DEmiRs are described in Table S1. miR-103a-3p and miR-423-5p were chosen as endogenous controls because variations in their expression levels were the lowest, and *p* values were the highest detected in the microarray analysis. Also, their reliability as endogenous controls were confirmed in qRT-PCR experiments once they presented stable expression in all the samples. Each sample was run in triplicate, and FC was calculated using the comparative CT method (2^−ΔΔCT^) [25]. We compared the miRNA expression levels of TT (*n* = 13) and NNT (*n* = 13) groups using paired Student *t*-test, *p* < 0.05, FDR < 0.05, and 2000 permutations. The statistical analyses were performed using TM4 MultiExperiment Viewer (MeV) 4.9 software, and boxplots were constructed using SPSS version 25.0.

### 2.5. Array Express Dataset for In Silico Analysis of miRNA Expression in PeC

ArrayExpress database, a repository for expression data from the European Bioinformatics Institute (EMBL-EBI), was accessed to obtain raw data from the E-MTAB-3087 dataset (https://www.ebi.ac.uk/arrayexpress/experiments/E-MTAB-3087/, accessed on 13 April 2021). This dataset includes miRNA expression levels from fresh TT and their matched NNT [12] of PeC patients. Each group was represented by a raw file (fastq format) with data from a pool of ten samples. Both TT and NNT gene expression files were downloaded and pre-processed as suggested by authors [12]. Cutadapt v.3.4 and miRge3.0 software were used for processing samples. All values are expressed in log_2_ (normalized counts +1) ratio between TT and NNT counts. We considered all ratio values above 1.0 as upregulated and all values below −1.0 as downregulated.

### 2.6. Pathway Enrichment Analysis of the Predicted Targets of the DEmiRs and Establishment of miRNA-mRNA Interaction Networks

The web tool Mienturnet [26] was used to access the miRTarBase 8.0 database (release date 15 September 2019) to search for experimentally validated target genes of the eight DEmiRs detected and validated in our study when comparing TT relative to NNT samples. The software Gephi v.0.9.2 was used to construct miRNA-mRNA interactions as network plots. Networks were constructed considering 100 top miRNA-mRNA interactions with a weak and strong functional level of evidence and FDR < 0.05 for downregulated and FDR < 0.1 for upregulated DEmiRs. The Reactome database was used for pathway enrichment analysis to search pathways related to miRNA targets with either weak or strong levels of evidence and FDR < 0.01. The Mienturnet tool allowed graphing of the enriched plot, where each dot represents one miRNA whose targets are included in a pathway. Each dot was colored by *p*-adjusted value and sized by gene ratio per pathway.

### 2.7. mRNA Expression Profiling by a High-Throughput Nanofluidic qRT-PCR Platform

We further explored the differentially expressed genes (DEGs) in all TT (*n* = 24) and NNT (*n* = 24) samples using a panel containing 83 genes of interest and seven endogenous control genes (Table S2). This panel was composed of genes targeted by the identified and validated DEmiRs using microarray and qRT-PCR, genes related to carcinogenesis, epithelial-mesenchymal transition (EMT), DNA damage response, cell cycle, and epigenetic disturbs, in addition to genes previously described as deregulated in PeC and other SSC.

Gene expression levels were evaluated by qRT-PCR using the nanofluidic platform Biomark HD System (Fluidigm, South San Francisco, CA, USA), according to the manufacturer’s instructions. A total of 45 ng RNA was used for cDNA synthesis (Fluidigm) followed by 10 cycles of pre-amplification reaction, which was performed using a pool of 90 Delta Gene™ assays (Fluidigm) representing all investigated genes at a final concentration of 500nM each. Assays were designed using the D3 Assay Design software (Fluidigm) containing Forward and Reverse primers. The pre-amplified cDNA was treated with Exonuclease I (New England BioLabs, Ipswich, MA, USA) and diluted 1:10.

Gene expression levels were quantified by qRT-PCR using the EvaGreen^®^ dye method (Bio-Rad Laboratories, Hercules, CA, USA) and were prepared according to the manufacturer’s instructions. Delta Gene™ Assays (final concentration of 5uM) and solutions were pipetted into the 96.96 Dynamic Array Integrated Fluidic Circuit (IFC) according to the manufacturer’s recommended pipetting map and then placed into the Juno IFC controller (Fluidigm) to load the samples and assays in the 96.96 Dynamic Array IFC. After this run, qRT-PCRs were conducted using the nanofluidic platform Biomark™ HD System Real-Time PCR (Fluidigm) according to the established protocol. The results were extracted from the Biomark Data Collection version 4.5.1 software and were analyzed using the Fluidigm Real-Time PCR Analysis version 4.3.1 software (Fluidigm). Obtained values were plotted individually for each gene, and only samples with CT lower than 24 were considered for analysis.

NormFinder, a Microsoft^®^ Excel add-in [27], was used to assess the stability of the expression levels of the seven endogenous control genes (*ACTB*, *B2M*, *GAPDH*, *GUSB*, *HPRT1*, *RPLP0*, *TFRC*) used as candidates to normalize qRT-PCR data. Based on each gene’s intra- and inter-group variations, this program can automatically determine the most stably expressed candidate reference genes and gene pairs in a sample. *ACTB* and *RPL0* were found to be the most stable endogenous genes in our samples. FC was calculated using the comparative CT method (2^−ΔΔCT^) [25]. The TM4 MeV 4.9 software was used to detect DEGs comparing TT and NNT groups using paired Student *t*-test, *p* < 0.05, FDR < 0.05, and 2000 permutations.

### 2.8. GEO Dataset for In Silico Analysis of Deregulated Gene Expression in PeC

The Gene Expression Omnibus (GEO), a publicly available genomics database at NCBI, was used to download the pre-processed data of the GSE57955 dataset (https://www.ncbi.nlm.nih.gov/geo/query/acc.cgi?acc=GSE57955, accessed on 17 April 2021). This dataset was obtained from a study that performed gene expression analysis using the Whole Human Genome 4 × 44 K microarray platform (Agilent Technologies), including 39 PeC samples and one pool including five autopsy glans from Brazilian patients [9]. To download and process data, we used the GEOquery, ggplot2, and dplyr packages for the R statistical software v. 4.0.2. All data were expressed in log_2_ signal ratio (Tumor/Normal glans). Ratio values above 1.0 were considered as upregulated and values below −1.0 as downregulated.

### 2.9. Gene Set Enrichment Analysis (GSEA) of Deregulated Genes in PeC

FC values were used to rank DEGs detected in the comparison between TT and NNT for GSEA analysis. We used the msigdbr package for the R software v. 4.0.2 to download the Gene Ontology (GO) database information for pathways and genes. The clusterProfiler package was used to perform the GSEA by comparing our gene list with all pathways with more than 10 genes and less than 500 genes. Enriched pathways with *p* < 0.05 were selected to represent a metabolic map and GSEA plots using the enrichplot package.

### 2.10. DNA Extraction and Human Papillomavirus (HPV) Detection

After paraffin removal using xylene, PeC tissues were digested with proteinase K/SDS 0.1% for 24 h, and DNA was obtained by phenol:chloroform extraction. DNA concentration was determined using a Nanodrop 2000 spectrophotometer (Thermo Fisher Scientific), and DNA samples were diluted to 50 ng/µL. DNA quality was assessed by amplifying a 110 bp fragment of the human β-globin gene using PCO_3_/PCO_4_ primers followed by analysis in 8% acrylamide gel electrophoresis [28]. The Inno-LiPA HPV Genotyping kit (Innogenetics, Gent, Oost-Vlaanderen, Belgium) was used for HPV DNA detection and genotyping as previously described [29]. This technology can discriminate 28 different HPV types by reverse blot hybridization.

### 2.11. Statistical Analyses

The correlation analyses between expression levels of nine miRNAs and 83 genes were performed by Pearson correlation test using SPSS version 25.0 separately for TT and NNT samples. All correlation values were plotted in R statistical software v. 4.0.2 using the corrplot, ggplot2, ggrepel, and ggpubr packages. All miRNA-mRNA expression levels combinations were plotted in a scatter plot to select pairs showing inverse correlation values between the two evaluated groups, which was determined by outliers of a linear distribution from the most negatively correlated R values (−1, −1) to the most positively correlated R values (1, 1) with a confidence interval equal to 99%.

The Receiver Operating Characteristic (ROC) curves were constructed using SPSS version 25.0, and the area under the curve (AUC) was calculated to analyze the accuracy of each DEmiR and DEG for distinguishing TT from NNT with specificity and sensitivity. For the eight miRNA-mRNA pairs identified in the correlation analyses, ROC curves analysis was performed to evaluate the diagnostic test ability of these pairs (using the ratio values of -delta CT miRNA and-delta CT mRNA).

## 3. Results

### 3.1. Patients, Treatment, and Follow Up

Between July 2015 and January 2018, 41 new cases of PeC were diagnosed at ICESP, of which 24 patients were included in the study. The reasons for the exclusion of the reminiscent 17 patients are detailed in Table S3.

Table 1 describes the clinical and histopathological data of all PeC patients (*n* = 24), and separately for the set of samples used for miRNA microarray analysis (*n* = 11), and the independent validation (*n* = 13) cohort. All specimens were classified as usual histological subtype of squamous cell carcinoma of the penis. The mean (SD) age at diagnosis was 61.8 (16.1) years old, and the median follow-up time was 39.8 months. Twenty patients (83.3%) presented a high risk for inguinal metastasis according to EAU classification [24]. Partial penectomy was carried out in 17 patients (70.8%), and inguinal and pelvic lymphadenectomy was performed in 16 (66.7%) and four (16.7%) patients, respectively. Eleven patients had inguinal metastasis confirmed by pathology, and one was classified as cN3. At the analysis time, ten patients (41.7%) had died from any cause, and seven (29.2%) due to PeC. HPV DNA was detected in tumors obtained from eight patients (33.3%). The most common type was HPV-16 (*n* = 3), however HPVs 11 (*n* = 1), 33 (*n* = 1), 35 (*n* = 1), 52 (*n* = 1), 58 (*n* = 1), 59 (*n* = 1) and 68 (*n* = 1) types were also detected.

### 3.2. miRNAs Expression Profiles, Interaction Networks of miRNA and Predicted Target Genes and Pathway Enrichment Analysis

**Figure 1:**
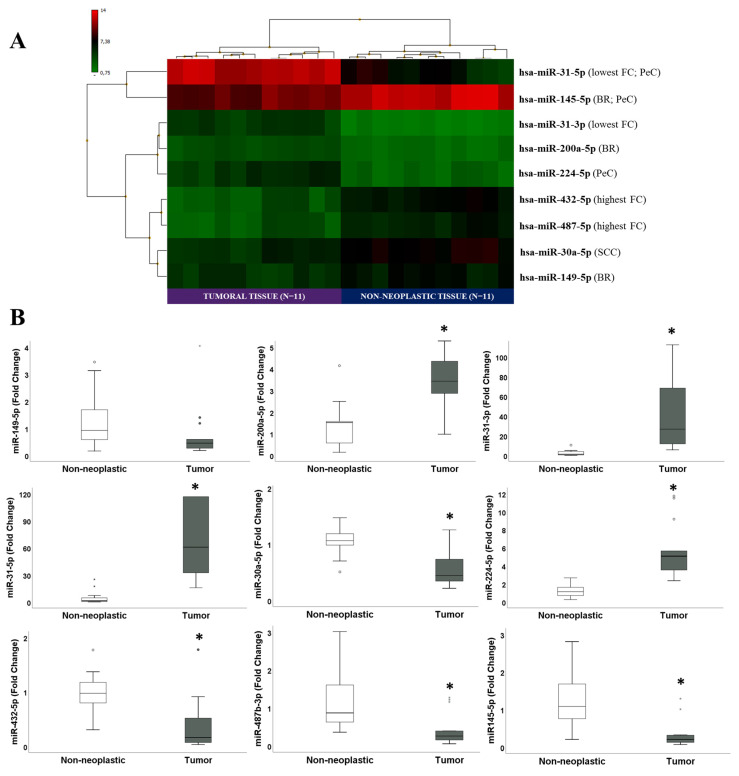
Differentially expressed miRNAs (DEmiRs) identified in the comparison between tumoral tissues (TT) and non-neoplastic tissues (NNT) by microarray analysis (*n* = 11) and chosen for validation in the independent cohort of penile cancer (PeC) patients (*n* = 13). (**A**) Hierarchical clustering analysis shows that the nine DEmiRs chosen for validation were able to cluster samples according to group. The reason for the selection of each DEmiR is indicated in parenthesis (FC: fold change; BR: Biological Relevance; SCC and PeC: previously described at Squamous Cell Carcinoma and penile cancer, respectively); (**B**) The box-plots represent the comparison of the expression levels of the nine selected DEmiRs in the TT (*n* = 13) relative to NNT (*n* = 13) in the independent validation set of samples; only miR-149-5p was not validated; * *p* < 0.05.

Initially, microarray analysis identified 69 DEmiRs when comparing TT (*n* = 11) relative to NNT (*n* = 11), among which 28 DEmiRs were found to be upregulated and 41 downregulated. The complete list of DEmiRs with respective FC and *p* values is depicted in Table S4. Figure 1A shows the criteria used to select the nine DEmiRs that were further validated by qRT-PCR and demonstrates that these were able to cluster samples according to group. Eight out of nine DEmiRs were validated in the independent set of samples (*n* = 13), as shown in Table 2 and Figure 1B. Only miR-149-5p was not validated.

We performed an external analysis of these eight DEmiRs using the E-MTAB-3087 dataset. The original study, from which this dataset was derived, compared miRNA expression levels of PeC tissues from ten patients with their matched non-cancerous adjacent tissues [12]. One upregulated DEmiR (miR-31-5p) and all four downregulated DEmiRs detected in our study (miR-30a-5p, miR-432-5p, miR-487b-3p and miR-145-5p) were in silico confirmed using this dataset (Table 3).

To investigate the accuracy in distinguishing TT from NNT, we analyzed ROC curves for the eight DEmiRs validated in our study, and we found that all four downregulated DEmiRs presented AUC > 0.89 (Figure S1), although none of the upregulated DEmiRs reached similar results.

**Figure 2:**
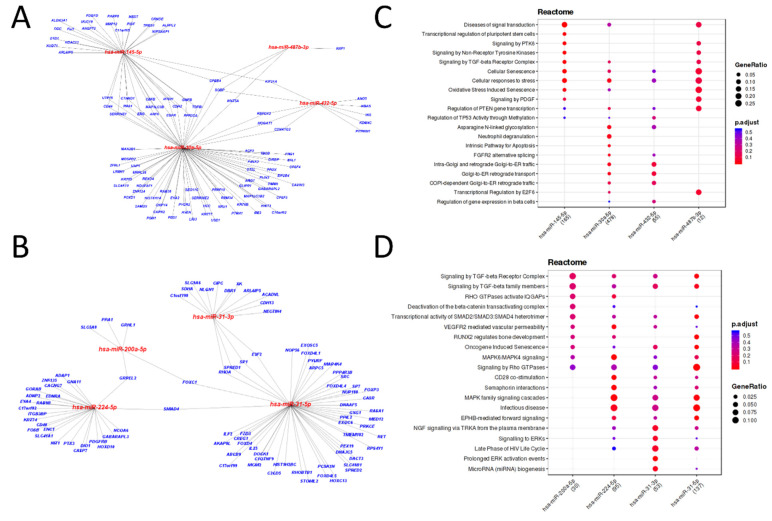
Experimentally validated targets of differentially expressed miRNAs (DEmiRs) of the comparison between tumoral x non-neoplastic tissues. (**A**,**B**) The miRTarBase 8.0 database supported information for networks representing miRNA and their experimentally validated target genes with weak and strong evidence levels for downregulated (**A**) or upregulated (**B**) DEmiRs; (**C**,**D**) Enrichment plots of Reactome pathways for experimentally validated miRNA targets with weak and strong evidence levels for downregulated (**C**) or upregulated (**D**) DEmiRs obtained from the Mienturnet tool. Each enrichment plot includes dots indicating miRNAs whose targets participate in the corresponding pathway. These dots are colored by *p*-adjusted value and sized by the gene ratio per pathway.

According to miRTarBase 8.0, we identified 38 and 39 experimentally validated target genes with a strong level of evidence for the down and upregulated DEmiRs, respectively. Interaction of the eight DEmiRs and respective target genes are represented as network plots in Figure 2A,B. Reactome database revealed that the target genes of the downregulated DEmiRs were grouped in pathways related to senescence and cellular response to stress (Figure 2C). Regarding the upregulated DEmiRs, their targets are involved in MAPK signaling and response to infectious disease pathways (Figure 2D). Results are shown as a dot plot representing the main enriched pathways for targets of each analyzed miRNA. Dots are colored by *p*-adjusted value and sized by gene ratio per pathway. Table S5 lists the top ten enriched pathways with a higher number of validated target genes of the DEmiRs with FDR < 0.01.

### 3.3. Deregulated mRNA Expression Profiling in PeC

**Figure 3:**
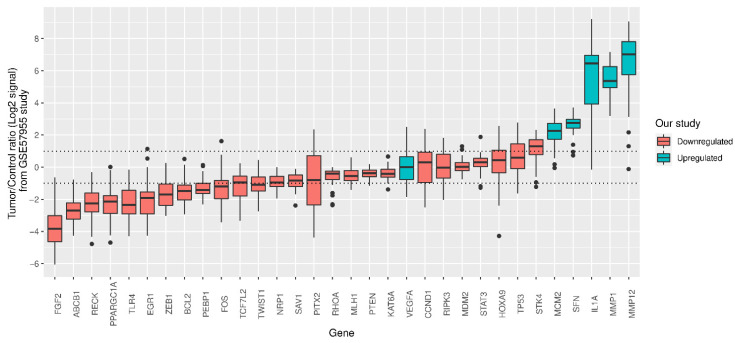
Gene expression ratios of log2 signal between penile cancer samples (*n* = 39) and a pool of five autopsy glans (control group) from the GSE57955 dataset. Thirty-two (out of 37) differentially expressed genes (DEGs) detected in our study were available in this dataset. All median values above 1.0 or below −1.0 were considered as differentially expressed in the GSE57955 study. Box plots for each gene were colored according to its regulation type in our study (red for downregulated genes and blue for upregulated genes). Five upregulated genes and ten downregulated genes from our study were confirmed in this dataset.

Regarding the expression levels of the 83 transcripts investigated in our gene panel, we identified 37 DEGs when TT was compared to NNT in all 24 PeC patients (Table 4). We observed that 31 DEGs were downregulated while only six were upregulated. *MMP1* was the most upregulated gene (FC = 28.0), followed by *IL1A* (FC = 13.4). The GSE57955 dataset was used for external analysis of the DEGs detected in our study. In the study from which this dataset was derived, 39 PeC samples were compared with five autopsy glans [9]. We found that 32 out of 37 DEGs described in our list were also available in their dataset. Therefore, we were able to in silico confirm five upregulated genes (*MCM2*, *SFN*, *IL1A*, *MMP1,* and *MMP12*) and 10 downregulated genes (*FGF2*, *ABCB1*, *RECK*, *PPARGC1A*, *TLR4*, *EGR1*, *ZEB1*, *BCL2*, *PEBP1,* and *FOS*) using this dataset (Figure 3).

**Figure 4:**
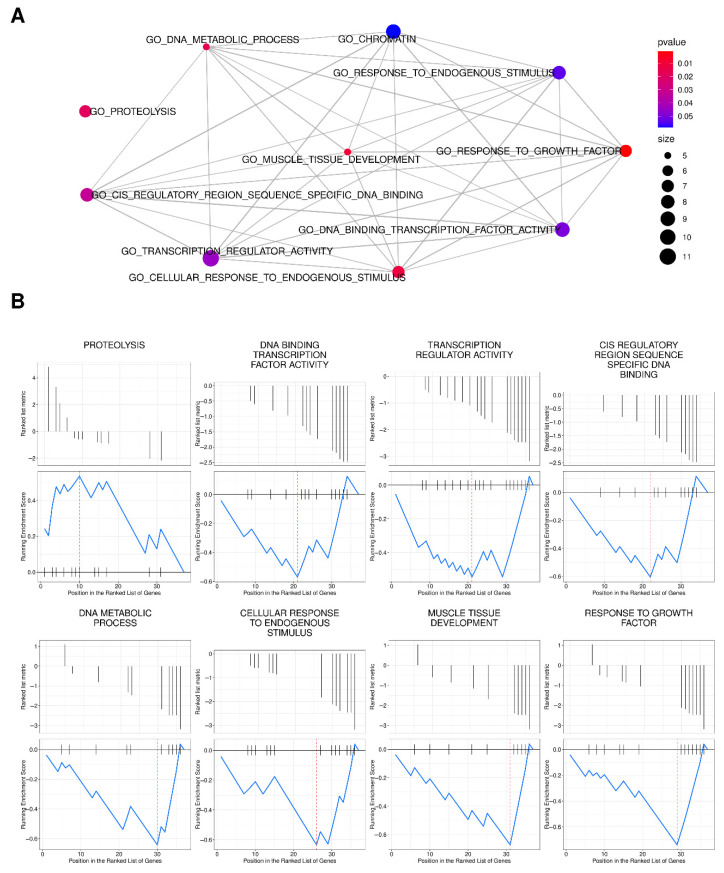
Gene Set Enrichment Analysis (GSEA) for Differentially Expressed Genes (DEGs) in the Gene Ontology (GO) database. (**A**) Biological network of the most represented GO terms, including the 37 detected DEGs in our study. Plots are represented as linked dots colored by *p*-value and sized by the number of involved genes; (**B**) GSEA plots indicate the DEGs’ distribution for each pathway. Blue lines represent the fluctuation of the Enrichment Score for each selected pathway indicating which one was upregulated (above the horizontal black bar) or downregulated (below the horizontal black bar).

GSEA analysis demonstrated that the identified DEGs are involved in the upregulation of the proteolysis pathway and downregulation of pathways related to the cellular response to endogenous stimulus, response to growth factors, and transcription regulator activity (*p* < 0.05) according to the GO database (Figure 4 and Table S6).

When performing the ROC curves for the 37 DEGs detected in our study, we observed that all six upregulated genes showed AUC > 0.77 (*IL1A*, *MCM2*, *MMP1*, *MMP12*, *SFN* and *VEGFA*) and presented good accuracy in discriminating TT from NNT groups as shown in Figure S2.

### 3.4. Penile Carcinogenesis Alters Expression Profiles of miRNA-mRNA Pairs

To integrate our data of miRNA and mRNA expression profiles and better understand miRNA-mRNA regulation, we searched for the experimentally validated transcripts in miRTarBase 8.0 that could be regulated by the DEmiRs found in the comparison between TT and NNT in our study. The DEmiRs detected in the initial microarray analysis with opposite expression levels in relation to any of the 37 DEGs (downregulated DEmiR and upregulated DEG or vice-versa) are listed in Table S7.

**Figure 5:**
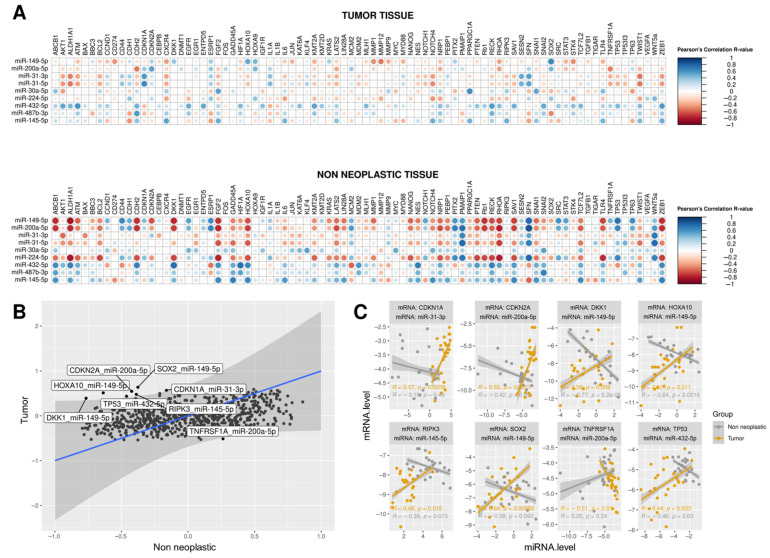
Pair-wise correlations between expression levels of 9 miRNAs and 83 genes in tumor tissues (TT) and non-neoplastic tissues (NNT) considering the total sample of 24 PeC patients. (**A**) Pearson correlation coefficients between expression levels of miRNAs and genes showed different profiles across TT and NNT samples. The size and color of each dot represent the Pearson correlation R-values for each miRNA-mRNA pair; (**B**) R-values were plotted, and eight miRNA-mRNA pairs out of the confidence interval of the linear equation were labeled and selected for further analysis. Each axis represents the correlation R-value for a specific tissue (TT or NNT); (**C**) miRNA and gene expression values (-delta CT) of TT (*n* = 24) and NNT (*n* = 24) were plotted for the eight miRNA-mRNA pairs selected in (**B**). For each graph, the R coefficient and *p* values of the Pearson Correlation test are indicated at the bottom.

We further plotted all Pearson correlation values between miRNA and mRNA expression levels for TT and NNT separately (Figure 5A), showing that expression levels of miRNAs and transcripts showed different profiles between groups. Next, a linear distribution allowed us to find the eight miRNA-mRNA pairs that changed correlation when comparing TT and NNT groups (Figure 5B,C). The most remarkable change was observed for the miR-432-5p-*TP53* pair, which showed a negative correlation in NNT and a positive correlation in TT (Figure 5C).

Finally, we performed the ROC curve analysis to investigate whether these miRNA-mRNA pairs could discriminate between TT and NNT groups. We observed that two pairs presented the AUC > 0.85 (miR-432-5p-*TP53* and miR-145-5p-*RIPK3*) and three AUC > 0.65 (miR-149-5p-*DKK1,* miR-149-5p-*SOX2,* and miR-149-5p-*HOXA10*; Figure S3) and they could be potential diagnostic biomarker candidates for PeC.

## 4. Discussion

We performed a prospective translational study aiming to identify a molecular signature for penile carcinogenesis based on miRNA and gene expression levels. Here, we describe some potential molecular markers and deregulated pathways that might help to clarify the mechanisms underlying PeC development, which may lay the basis for future therapeutic strategies.

The molecular basis of penile carcinogenesis is complex and not fully understood. High-risk (HR) HPV infections, especially HPV-16 infections, respond for approximately 30% of PeC cases [30], and may be prevented by vaccination. The role of HR-HPV E6 and E7 proteins in the deregulation of pRB and p53 leading to PeC has been investigated [7,8]. Nevertheless, other genes, proteins, and pathways functionally deregulated in non-HPV-associated PeC have also been explored, including EGFR [31], Wnt [32], MAPK/ERK, and PI3K-AKT-mTOR pathways [7,8].

Non-coding miRNAs have been extensively studied and have been shown to impact the development and metastasis of malignant tumors [33,34]. Deregulated expression of miRNAs may upregulate the expression of oncogenes or downregulate the expression of tumor suppressor genes, as well as play a role in other mechanisms involved in carcinogenesis [33,34]. In 2015, Zhang et al. published the first study regarding the identification of deregulated miRNAs detected by next-generation sequencing associated with penile carcinogenesis and suggested networks of predicted target genes and signaling pathways that could be involved in malignant transformation [12].

In our study, we also used a high-throughput technology to unravel potential miRNAs involved with penile carcinogenesis. Initially, microarray analysis identified 69 DEmiRs when comparing 11 paired samples (TT vs. NNT) of PeC patients. We were able to validate eight out of nine DEmiRs (miR-432-5p; miR-487b-3p; miR-145-5p; miR-30a-5p; miR-200a-5p; miR-224-5p; miR-31-3p and miR-31-5p) by qRT-PCR in an independent set of 13 PeC samples. In addition, we externally confirmed one upregulated DEmiR (miR-31-5p) and all four downregulated DEmiRs (miR-30a-5p, miR-432-5p, miR-487b-3p, and miR-145-5p) detected in our study using the original dataset (E-MTAB-3087) obtained from Zhang et al. [12], corroborating the importance of these DEmiRs in penile carcinogenesis.

Our results are in accordance with some findings of Kuasne et al. (2017) who evaluated miRNA expression profiles by microarray comparing TT (*n* = 23) and NNT (*n* = 12) in a Brazilian sample of PeC patients [14]; ten out of the 81 DEmiRs identified by them were also detected in our microarray analysis among which six were downregulated (let-7c-5p, miR-134-5p, miR-139-3p, miR-139-5p, miR-145-5p and miR-574-3p) and four upregulated (miR-183-5p, miR-18a-5p, miR-224-5p and miR-31-5p). Moreover, two upregulated (miR-224-5p and miR-31-5p) and one downregulated DEmiR (miR-145-5p) were validated by qRT-PCR in both studies [14]. Furthermore, although not selected for validation, we found that miR-99a-5 was downregulated in the comparison of TT vs. NNT samples (Table S4), corroborating a recent study in HPV-negative usual subtype of PeC tumors [18].

We additionally demonstrated that four downregulated DEmiRs (miR-30a-5p, miR-432-5p, miR-487b-3p, and miR-145-5p) presented good accuracy by estimating the AUC, and were capable of discriminating TT from NNT.

miRTarBase 8.0 database enabled identifying a list of the currently validated target genes for the identified down and upregulated DEmiRs and constructing regulatory networks. We showed a complex network with several genes being regulated by the DEmiRs, with more than one DEmiR regulating some genes, among which some interactions were confirmed in our study. Regarding pathway enrichment analysis, experimentally validated targets of the downregulated DEmiRs are involved in pathways related to senescence and cellular response to stress. Concerning the upregulated DEmiRs, targets genes are enriched in MAPK family signaling and response to infectious disease pathways. These results provide additional insights into altered molecular pathways involved in the PeC tumorigenesis.

We identified 37 DEGs when comparing TT and NNT by qRT-PCR. Twenty-five of these are validated targets from at least one of the 69 DEmiRs identified by us. The highest upregulation was observed for *MMP1* (FC = 28.0) and *IL1A* (FC = 13.4). The biological functions of the 37 deregulated genes, employing both GO database and GSEA analysis, show that these genes are enriched in proteolysis and also in pathways related to cellular response to endogenous stimulus, response to growth factors, and transcription regulator activity. Once gene expression has been analyzed using a cancer-related gene panel, our GSEA analysis were restricted to only an ontology analysis due to the lack of urological disease-related gene expression datasets. An imputation with other broad datasets could also produce a result bias.

The original dataset (GSE57955) of Kuasne et al. [9] was accessed for external analysis of the DEGs detected in our work. We found that 32 out of 37 DEGs described in our list were also available in their dataset. Therefore, we could in silico confirm five upregulated (*IL1A*, *MCM2*, *MMP1*, *MMP12,* and *SFN*) and ten downregulated genes (*ABCB1*, *BCL2*, *EGR1*, *FGF2*, *FOS*, *PEBP1*, *PPARGC1A*, *RECK*, *TLR4*, and *ZEB1*). *VEGFA* was the only upregulated gene from our study that was not confirmed by this external dataset. This discrepancy might be due to differences in the sample composition. In the original dataset from Kuasne et al. [9], the TT and NNT samples were not paired and three PeC patients were classified as histological subtype different from usual SCC. In the analysis of ROC curves, we found that all the six upregulated genes detected by our study (*IL1A*, *MCM2*, *MMP1*, *MMP12*, *SFN* and *VEGFA*) presented good accuracy to discriminate TT and NNT groups. Interestingly, we could identify some potential miRNA-mRNA regulations in our study that could be potential biomarkers candidates in PeC. Five of these upregulated DEGs are described as experimentally validated targets (according to miRTarBase 8.0 database) of two downregulated DEmiRs that were also validated and showed good accuracy to discriminate TT and NNT in the present study (miR-145-5p and *MCM2*, *MMP1*, *MMP12* and *VEGFA*; and miR-30a-5p and *IL1A*). In fact, miR-145-5p has also been described as deregulated in PeC in previous studies [12,14], and the upregulation of *MMP1* and *MMP12* were validated by qRT-PCR by Kuasne et al. (2017) in PeC patients, also showing good accuracy in distinguishing TT from NNT (AUC of 0.923 and 0.865, respectively) [14]. The overexpression of *MMP1* and *MMP12* have been associated with the development of tumors in other anatomical regions, including esophageal SCC and head and neck SCC (HNSCC) [35]. *MCM2* and *VEGFA*, also targets of miR-145-5p, were overexpressed in our tumor samples. *MCM2* has been associated with prognostic factors in PeC, whereas *VEGFA* was shown overexpressed in oral SCC [36,37].

Downregulation of miR-30a-5p, a tumor suppressor miRNA, has been previously implicated in HNSCC development [38]. One of its targets, *IL1A,* was also shown upregulated in cervical carcinoma [39]. The overexpression of *IL1A* can, in turn, stimulate the transcription of growth factors such as *MMP1* [40] or *VEGFA* [41], both found upregulated in our tumor samples, possibly creating a proper environment for carcinogenesis.

miR-31 has been found altered in several types of cancers and was shown to influence various cellular functions, including migration, proliferation, viability, metastasis, apoptosis, and sensitivity to therapies [42]. Its functional role is complex and may act as a tumor suppressor or oncogene in different types of tumors in a context-dependent manner [42]. In our study, miR-31-5p presented the highest FC, and it has been previously implicated in the tumorigenesis of PeC [14]. miR-31-5p inhibits the tumor suppressor *LATS2* gene, promoting oral SCC progression by inhibiting the Hippo signaling pathway [43], a transcript that we also found downregulated in our analysis. *MLH1* was also downregulated in our casuistic and it has been demonstrated to be a direct target of miR-31-5p in non-small cell lung cancer cell lines [44].

We explored the regulation of nine miRNA and 83 mRNA expression profiles investigated in the present study by an integrative analysis of miRNA-mRNA correlation instead of only comparing the mean values of miRNA and mRNA expression levels between groups. We revealed the disruption in the dynamics of the regulation of miRNA-mRNA pairs in PeC showing that eight pairs changed the correlation patterns during tumor development, i.e., negative correlations that changed to positive or vice-versa when comparing NNT and TT groups. The most remarkable pair was miR-432-5p-*TP53*, for which we showed a negative correlation between miRNA-mRNA observed in NNT that was altered to positive in TT. According to the miRTarBase 8.0 database, the only previous validated pair regulation was miR-149-5p-*HOXA10*. miR-149-5p was shown to be downregulated in HPV-positive cervical cancer [45], and *HOXA10* has been shown to play a role in oral SCC impacting proliferation, migration, and invasion [46]. However, this is the first study to demonstrate that altered interaction of miR-149-5p-*HOXA10* might be implicated in penile carcinogenesis. miR-149-5p has also changed the correlation pattern with *DKK1* and *SOX2* transcripts, both shown overexpressed in HNSCC [47,48]. The other seven miRNA-mRNA pairs in PeC have been reported here for the first time.

Finally, we demonstrated that five out of these eight disrupted miRNA-mRNA pairs (miR-432-5p-*TP53*, miR-145-5p-*RIPK3*, miR-149-5p-*HOXA10*, miR-149-5p-*DKK1,* and miR-149-5p-*SOX2*) showed accuracy in discriminating TT and NNT groups and could be further investigated as potential diagnostic biomarker candidates for PeC. Taken together, the present work described novel molecular signatures for PeC based not only on the disruption of miRNA and mRNA expression levels themselves, but also in miRNA-mRNA pairs that changed regulation during the penile carcinogenesis process.

It is important to mention that this study was limited by the small sample size mainly related to the rarity of this disease, which precludes cohorts with a high sample number. This might have impacted our validation by qRT-PCR in an independent cohort. Furthermore, we did not find any public datasets with miRNA and transcripts expression levels in PeC for in silico evaluation of the disruption of miRNA-mRNA regulation proposed by this work. Then, further studies with larger PeC cohorts are required to confirm our findings and to functionally validate the new miRNAs-mRNAs interactions. Protein expression levels must also be evaluated to define these as potential biomarkers and targets for new therapeutics in PeC.

An additional limitation was that more than 80% of the studied cases were classified as a high-risk disease. This might be because our hospital is a Tertiary Referral Care Center and we generally receive patients in advanced stages of the disease. Moreover, higher stages of PeC have been associated with low socioeconomic status [49], and 86% of our patients reached only primary school (data not shown). In this sense, we also acknowledge that potential dysregulation of inflammation-related genes included in our proposed molecular signature (e.g., *IL1A*, *MCM2*, *MMP1*, *MMP12,* and *VEGFA*) might be due to the inflammation favoring tumor maintenance and progression [50,51].

On the other hand, it is noteworthy that samples and clinical data were prospectively collected, and patients were uniformly treated according to the international guidelines [24]. We also confirmed results from microarray by qRT-PCR in an independent cohort of patients and in silico analysis from an external cohort. Another key strength of the present study was the novel interaction between miRNAs and transcript expression levels suggesting regulations by miRNAs not previously reported.

## 5. Conclusions

We described a molecular signature based on eight miRNAs (miR-432-5p; miR-487b-3p; miR-145-5p; miR-30a-5p; miR-200a-5p; miR-224-5p; miR-31-3p and miR-31-5p) and 37 gene expression levels that was associated to penile carcinogenesis. We also described changes in the regulation of eight miRNA-mRNA pairs during tumor development. Taken together, our findings contribute to a better understanding of the regulatory roles of miRNAs and altered transcripts in the carcinogenesis process of PeC, suggesting potential molecular biomarkers and deregulated pathways involved in the mechanisms of penile carcinogenesis.

## Figures and Tables

**Table 1:** Clinical and histopathological characteristics of PeC patients for each set of samples.

Patients	Total Sample *n* (%)	Microarray Sample *n* (%)	Validation Sample *n* (%)
Number of patients	24	11	13
Age at surgery-Mean (SD) years old	61.8 (16.1)	61.6 (14.2)	61.9 (18.2)
Follow up-Median (range) months	39.8 (2–68)	47.5 (8–62)	34.9 (2–61)
Smoking history	14 (58.3)	8 (72.7)	6 (46.1)
cT			
cT1	3 (12.5)	2 (18.2)	1 (7.7)
cT2	13 (5.2)	4 (36.4)	9 (69.2)
cT3	8 (33.3)	5 (45.4)	3 (23.1)
cN			
cN0	9 (37.5)	5 (45.4)	4 (30.8)
cN1	8 (33.3)	3 (27.3)	5 (38.4)
cN2	4 (16.7)	3 (27.3)	1 (7.7)
cN3	3 (12.5)	0 (0)	3 (23.1)
Penectomy			
Partial	17 (70.8)	7 (63.6)	10 (76.9)
Total	7 (29.2)	4 (36.4)	3 (23.1)
Grade			
I	3 (12.5)	2 (18.2)	1 (7.7)
II	13 (54.2)	6 (54.5)	7 (53.9)
III	8 (33.3)	3 (27.3)	5 (38.4)
T Stage			
pT1	4 (16.6)	3 (27.2)	1 (7.7)
pT2	13 (54.2)	4 (36.4)	9 (69.2)
pT3	7 (29.2)	4 (36.4)	3 (23.1)
Inguinal Lymphadenectomy	16 (66.7)	7 (63.6)	9 (69.2)
Pelvic Lymphadenectomy	4 (16.7)	2 (18.1)	2 (15.4)
Lymph node metastasis	12 (50.0)	6 (54.5)	6 (46.2)
Presence of HPV infection	8 (33.3)	3 (27.3)	5 (38.4)
Tumor Size-Mean (SD) cm	4.75 (2.27)	4.93 (2.50)	4.59 (2.15)
Lymphovascular invasion	6 (25.0)	3 (27.3)	3 (23.1)
Perineural invasion	10 (41.7)	4 (36.4)	6 (46.2)
Group Risk (EAU)			
Low	1 (4.2)	1 (9.1)	0 (0)
Intermediate	3 (12.5)	2 (18. 2)	1 (7.7)
High	20 (83.3)	8 (72.7)	12 (92.3)

PeC: penile cancer; *n*: number of individuals; SD: standard deviation; HPV: human papillomavirus; EAU: European Association of Urology.

**Table 2:** Nine DEmiRs identified in the comparison between TT and NNT by microarray analysis (*n* = 11) and chosen for validation in the independent cohort of PeC patients (*n* = 13) by qRT-PCR.

Regulation	DEmiR	Microarray		qRT-PCR		
		FC	FDR	FC	SD	FDR
Downregulated	miR-432-5p	0.07	0.0048 *	0.41	0.51	0.004 *
	miR-487b-3p	0.12	0.0079 *	0.44	0.45	0.004 *
	miR-145-5p	0.18	0.0088 *	0.33	0.37	0.005 *
	miR-30a-5p	0.21	0.0067 *	0.57	0.32	0.002 *
	miR-149-5p	0.29	0.0308 *	0.79	1.04	0.097
Upregulated	miR-200a-5p	2.93	0.0048 *	3.48	1.28	0.004 *
	miR-224-5p	6.59	0.0083 *	5.70	3.19	0.001 *
	miR-31-3p	12.38	0.0002 *	41.62	41.43	0.004 *
	miR-31-5p	38.32	0.0048 *	122.28	127.75	0.001 *

DEmiR: differentially expressed miRNA; TT: tumor tissue; NNT: non-neoplastic tissue; *n*: number of individuals; PeC: penile cancer; FC: fold change; SD: standard deviation; FDR: false discovery rate; qRT-PCR: quantitative Reverse Transcription Polymerase Chain Reaction; * *p* < 0.05.

**Table 3:** miRNA expression analysis represented by log_2_ (normalized counts + 1) ratio between PeC samples and their matched adjacent non-cancerous tissues from the E-MTAB-3087 dataset.

Regulation in Our Study	miRNA	E-MTAB-3087 Dataset
		log2 (Normalized Counts + 1)
Downregulated	miR-30a-5p *	−8.165
	miR-432-5p *	−7.947
	miR-487b-3p *	−6.236
	miR-145-5p *	−3.425
Upregulated	miR-200a-5p	−0.551
	miR-224-5p	−5.815
	miR-31-3p	−3.342
	miR-31-5p *	2.914

PeC: penile cancer; * Differentially expressed miRNAs validated by qRT-PCR in our study and detected in the E-MTAB-3087 dataset.

**Table 4:** DEGs detected in the comparison of tumor tissues (*n* = 24) and non-neoplastic tissues (*n* = 24) of PeC patients by qRT-PCR.

Reg	DEG	FC	FDR	DEG	FC	FDR	DEG	FC	FDR
Down	*ABCB1*	0.24	<0.001 *	*LATS2*	0.58	0.004 *	*RIPK3*	0.53	<0.001 *
	*ALDH1A1*	0.09	<0.001 *	*MDM2*	0.71	0.003 *	*SAV1*	0.45	<0.001 *
	*BCL2*	0.31	<0.001 *	*MLH1*	0.77	0.007 *	*STAT3*	0.66	<0.001 *
	*CCND1*	0.61	0.007 *	*NANOG*	0.33	0.005 *	*STK4*	0.65	0.002 *
	*EGR1*	0.19	<0.001 *	*NRP1*	0.48	<0.001 *	*TCF7L2*	0.36	<0.001 *
	*FGF2*	0.18	<0.001 *	*PEBP1*	0.53	<0.001 *	*TLR4*	0.28	<0.001 *
	*FOS*	0.18	<0.001 *	*PITX2*	0.30	<0.001 *	*TP53*	0.57	0.003 *
	*HOXA9*	0.51	0.003 *	*PPARGC1A*	0.11	<0.001 *	*TWIST1*	0.18	<0.001 *
	*KAT6A*	0.47	<0.001 *	*PTEN*	0.55	<0.001 *	*ZEB1*	0.23	<0.001 *
	*KLF4*	0.22	<0.001 *	*RECK*	0.24	<0.001 *			
	*KMT2A*	0.40	<0.001 *	*RHOA*	0.66	<0.001 *			
Up	*IL1A*	13.39	<0.001 *	*MMP1*	28.00	<0.001 *	*SFN*	4.26	0.004 *
	*MCM2*	2.14	<0.001 *	*MMP12*	9.97	<0.001 *	*VEGFA*	2.06	<0.001 *

DEGs: differentially expressed genes; *n*: number of individuals; PeC: penile cancer; Reg: regulation; FC: fold change; FDR: false discovery rate; qRT-PCR: quantitative Reverse Transcription Polymerase Chain Reaction; * *p* < 0.05.

## Data Availability

Microarray datasets are available on the Gene Expression Omnibus (GEO) database (accession number GSE172095).

## Supplementary Materials

The following are available online at https://www.mdpi.com/article/10.3390/cancers13194745/s1. Figure S1. ROC curves of the eight differentially expressed miRNAs (DEmiRs) validated by qRT-PCR. All downregulated DEmiRs (miR-30a-5p, miR-432-5p, miR-487b-3p and miR-145-5p) demonstrated to have good accuracy to discriminate tumoral from non-neoplastic tissues in PeC patients; ROC: receiver operating characteristic; AUC: area under the curve. Figure S2. ROC curves of differentially expressed genes (DEGs) that demonstrated to have good accuracy to discriminate tumoral from non-neoplastic tissues (*IL1A*, *MCM2*, *MMP1*, *MMP12*, *SFN* and *VEGFA*) in PeC patients; ROC: receiver operating characteristic; AUC: area under the curve. Figure S3. Eight miRNA-mRNA pairs disrupted in the penile carcinogenesis and their respective ROC curves to analyze accuracy in discriminating tumoral from non-neoplastic tissues; ROC: receiver operating characteristic; AUC: area under the curve. Table S1. Taqman^®^ Advanced miRNA Assays of the differentially expressed miRNAs (DEmiRs) selected for validation by qRT-PCR. Table S2. Complete list of the genes included in the panel (83 genes of interest and seven endogenous controls) used to evaluate transcript expression levels in all tumoral (*n* = 24) and non-neoplastic (*n* = 24) tissues from PeC patients. Table S3. Total number of PeC patients included in the study and reasons for exclusion of 17 patients. Table S4. List of 69 DEmiRs identified by microarray analysis to discriminate between TT (*n* = 11) and N*N*T (*n* = 11) tissues. Table S5. Top ten enriched pathways of the experimentally validated target genes (weak or strong functional evidence) of the DEmiRs detected in the comparison between tumoral (*n* = 24) and non-neoplastic (*n* = 24) tissues according to miRTarBase 8.0 database. Table S6. List of Gene Ontology (GO) enriched pathways of differentially expressed genes detected by Biomark HD Fluidigm platform in the comparison between tumoral (*n* = 24) and non-neoplastic tissues (*n* = 24). Table S7. List of the 69 DEmiRs detected in the comparison of tumoral and non-neoplastic tissues in the microarray analysis with opposite expression levels relative to that of the 37 DEGs described as experimentally validated according to miRTarBase 8.0.

## References

[1] Christodoulidou M., Sahdev V., Houssein S., Muneer A. (2015). Epidemiology of penile cancer. Curr. Probl. Cancer.

[2] Sung H., Ferlay J., Siegel R.L., Laversanne M., Soerjomataram I., Jemal A., Bray F. (2021). Global Cancer Statistics 2020: GLOBOCAN Estimates of Incidence and Mortality Worldwide for 36 Cancers in 185 Countries. CA Cancer J. Clin..

[3] Coelho R.W.P., Pinho J.D., Moreno J.S., Garbis D., do Nascimento A.M.T., Larges J.S., Calixto J.R.R., Ramalho L.N.Z., da Silva A.A.M., Nogueira L.R. (2018). Penile cancer in Maranhao, Northeast Brazil: The highest incidence globally?. BMC Urol..

[4] Mulherkar R., Hasan S., Wegner R.E., Verma V., Glaser S.M., Kalash R., Beriwal S., Horne Z.D. (2019). National patterns of care for early-stage penile cancers in the United States: How is radiation and brachytherapy utilized?. Brachytherapy.

[5] Ornellas A.A., Kinchin E.W., Nobrega B.L., Wisnescky A., Koifman N., Quirino R. (2008). Surgical treatment of invasive squamous cell carcinoma of the penis: Brazilian National Cancer Institute long-term experience. J. Surg. Oncol..

[6] Gopman J.M., Djajadiningrat R.S., Baumgarten A.S., Espiritu P.N., Horenblas S., Zhu Y., Protzel C., Pow-Sang J.M., Kim T., Sexton W.J. (2015). Predicting postoperative complications of inguinal lymph node dissection for penile cancer in an international multicentre cohort. BJU Int..

[7] Thomas A., Necchi A., Muneer A., Tobias-Machado M., Tran A.T.H., Van Rompuy A.S., Spiess P.E., Albersen M. (2021). Penile cancer. Nat. Rev. Dis. Primers.

[8] Emmanuel A., Nettleton J., Watkin N., Berney D.M. (2019). The molecular pathogenesis of penile carcinoma-current developments and understanding. Virchows Arch..

[9] Kuasne H., Colus I.M., Busso A.F., Hernandez-Vargas H., Barros-Filho M.C., Marchi F.A., Scapulatempo-Neto C., Faria E.F., Lopes A., Guimaraes G.C. (2015). Genome-wide methylation and transcriptome analysis in penile carcinoma: Uncovering new molecular markers. Clin. Epigenet..

[10] Barzon L., Cappellesso R., Peta E., Militello V., Sinigaglia A., Fassan M., Simonato F., Guzzardo V., Ventura L., Blandamura S. (2014). Profiling of expression of human papillomavirus-related cancer miRNAs in penile squamous cell carcinomas. Am. J. Pathol..

[11] Munoz J.J., Drigo S.A., Barros-Filho M.C., Marchi F.A., Scapulatempo-Neto C., Pessoa G.S., Guimaraes G.C., Trindade Filho J.C., Lopes A., Arruda M.A. (2015). Down-Regulation of SLC8A1 as a Putative Apoptosis Evasion Mechanism by Modulation of Calcium Levels in Penile Carcinoma. J. Urol..

[12] Zhang L., Wei P., Shen X., Zhang Y., Xu B., Zhou J., Fan S., Hao Z., Shi H., Zhang X. (2015). MicroRNA Expression Profile in Penile Cancer Revealed by Next-Generation Small RNA Sequencing. PLoS ONE.

[13] Hartz J.M., Engelmann D., Furst K., Marquardt S., Spitschak A., Goody D., Protzel C., Hakenberg O.W., Putzer B.M. (2016). Integrated Loss of miR-1/miR-101/miR-204 Discriminates Metastatic from Nonmetastatic Penile Carcinomas and Can Predict Patient Outcome. J. Urol..

[14] Kuasne H., Barros-Filho M.C., Busso-Lopes A., Marchi F.A., Pinheiro M., Munoz J.J., Scapulatempo-Neto C., Faria E.F., Guimaraes G.C., Lopes A. (2017). Integrative miRNA and mRNA analysis in penile carcinomas reveals markers and pathways with potential clinical impact. Oncotarget.

[15] Peta E., Cappellesso R., Masi G., Sinigaglia A., Trevisan M., Grassi A., Di Camillo B., Vassarotto E., Fassina A., Palu G. (2017). Down-regulation of microRNA-146a is associated with high-risk human papillomavirus infection and epidermal growth factor receptor overexpression in penile squamous cell carcinoma. Hum. Pathol..

[16] Marchi F.A., Martins D.C., Barros-Filho M.C., Kuasne H., Busso Lopes A.F., Brentani H., Trindade Filho J.C.S., Guimaraes G.C., Faria E.F., Scapulatempo-Neto C. (2017). Multidimensional integrative analysis uncovers driver candidates and biomarkers in penile carcinoma. Sci. Rep..

[17] Pinho J.D., Silva G.E.B., Teixeira Junior A.A.L., Belfort M.R.C., Mendes J.M., Cunha I.W.D., Quintana L.G., Calixto J.R.R., Nogueira L.R., Coelho R.W.P. (2020). MIR-107, MIR-223-3P and MIR-21-5P Reveals Potential Biomarkers in Penile Cancer. Asian Pac. J. Cancer Prev..

[18] Ayoubian H., Heinzelmann J., Holters S., Khalmurzaev O., Pryalukhin A., Loertzer P., Heinzelbecker J., Lohse S., Geppert C., Loertzer H. (2021). miRNA Expression Characterizes Histological Subtypes and Metastasis in Penile Squamous Cell Carcinoma. Cancers.

[19] Silva J.D., Nogueira L., Coelho R., Deus A., Khayat A., Marchi R., Oliveira E., Santos A.P.D., Cavalli L., Pereira S. (2021). HPV-associated penile cancer: Impact of copy number alterations in miRNA/mRNA interactions and potential druggable targets. Cancer Biomark..

[20] Pinho J.D., Silva G.E.B., Teixeira Júnior A.A.L., Belfort M.R.D.C., Mendes J.M.M., Calixto J.D.R.R., Nogueira L.R., Burbano R.R., Khayat A.S. (2021). Downregulation of miR-145 is associated with perineural invasion in penile carcinoma. Transl. Androl. Urol..

[21] Fabian M.R., Sonenberg N. (2012). The mechanics of miRNA-mediated gene silencing: A look under the hood of miRISC. Nat. Struct. Mol. Biol..

[22] Friedman R.C., Farh K.K.H., Burge C.B., Bartel D.P. (2008). Most mammalian mRNAs are conserved targets of microRNAs. Genome Res..

[23] Brierley J.D., Gospodarowicz M.K., Wittekind C. (2016). TNM Classification of Malignant Tumors.

[24] Hakenberg O.W., Compérat E., Minhas S., Necchi A., Protzel C., Watkin N. The European Association of Urology (EAU) Guidelines on Penile Cancer. http://uroweb.org/guideline/penile-cancer/#10.

[25] Livak K.J., Schmittgen T.D. (2001). Analysis of relative gene expression data using real-time quantitative PCR and the 2(-Delta Delta C(T)) Method. Methods.

[26] Licursi V., Conte F., Fiscon G., Paci P. (2019). MIENTURNET: An interactive web tool for microRNA-target enrichment and network-based analysis. BMC Bioinform..

[27] Andersen C.L., Jensen J.L., Orntoft T.F. (2004). Normalization of real-time quantitative reverse transcription-PCR data: A model-based variance estimation approach to identify genes suited for normalization, applied to bladder and colon cancer data sets. Cancer Res..

[28] Saiki R.K., Gelfand D.H., Stoffel S., Scharf S.J., Higuchi R., Horn G.T., Mullis K.B., Erlich H.A. (1988). Primer-directed enzymatic amplification of DNA with a thermostable DNA polymerase. Science.

[29] Kleter B., van Doorn L.J., Schrauwen L., Molijn A., Sastrowijoto S., ter Schegget J., Lindeman J., ter Harmsel B., Burger M., Quint W. (1999). Development and clinical evaluation of a highly sensitive PCR-reverse hybridization line probe assay for detection and identification of anogenital human papillomavirus. J. Clin. Microbiol..

[30] de Sanjose S., Serrano B., Tous S., Alejo M., Lloveras B., Quiros B., Clavero O., Vidal A., Ferrandiz-Pulido C., Pavon M.A. (2018). Burden of Human Papillomavirus (HPV)-Related Cancers Attributable to HPVs 6/11/16/18/31/33/45/52 and 58. JNCI Cancer Spectr..

[31] Chaux A., Munari E., Katz B., Sharma R., Lecksell K., Cubilla A.L., Burnett A.L., Netto G.J. (2013). The epidermal growth factor receptor is frequently overexpressed in penile squamous cell carcinomas: A tissue microarray and digital image analysis study of 112 cases. Hum. Pathol..

[32] Arya M., Thrasivoulou C., Henrique R., Millar M., Hamblin R., Davda R., Aare K., Masters J.R., Thomson C., Muneer A. (2015). Targets of Wnt/ß-Catenin Transcription in Penile Carcinoma. PLoS ONE.

[33] Svoronos A.A., Engelman D.M., Slack F.J. (2016). OncomiR or Tumor Suppressor? The Duplicity of MicroRNAs in Cancer. Cancer Res..

[34] Saliminejad K., Khorram Khorshid H.R., Soleymani Fard S., Ghaffari S.H. (2019). An overview of microRNAs: Biology, functions, therapeutics, and analysis methods. J. Cell. Physiol..

[35] Gobin E., Bagwell K., Wagner J., Mysona D., Sandirasegarane S., Smith N., Bai S., Sharma A., Schleifer R., She J.X. (2019). A pan-cancer perspective of matrix metalloproteases (MMP) gene expression profile and their diagnostic/prognostic potential. BMC Cancer.

[36] May M., Burger M., Otto W., Hakenberg O.W., Wieland W.F., May D., Hofstadter F., Gotz S., Niessl N., Fritsche H.M. (2013). Ki-67, mini-chromosome maintenance 2 protein (MCM2) and geminin have no independent prognostic relevance for cancer-specific survival in surgically treated squamous cell carcinoma of the penis. BJU Int..

[37] Nayak S., Goel M.M., Chandra S., Bhatia V., Mehrotra D., Kumar S., Makker A., Rath S.K., Agarwal S.P. (2012). VEGF-A immunohistochemical and mRNA expression in tissues and its serum levels in potentially malignant oral lesions and oral squamous cell carcinomas. Oral. Oncol..

[38] Saleh A.D., Cheng H., Martin S.E., Si H., Ormanoglu P., Carlson S., Clavijo P.E., Yang X., Das R., Cornelius S. (2019). Integrated Genomic and Functional microRNA Analysis Identifies miR-30-5p as a Tumor Suppressor and Potential Therapeutic Nanomedicine in Head and Neck Cancer. Clin. Cancer Res..

[39] Song Z., Lin Y., Ye X., Feng C., Lu Y., Yang G., Dong C. (2016). Expression of IL-1alpha and IL-6 is Associated with Progression and Prognosis of Human Cervical Cancer. Med. Sci. Monit..

[40] Inoue A., Obayashi K., Sonoda Y., Nakamura A., Ueno T., Kuhara S., Tashiro K. (2017). Regulation of matrix metalloproteinase-1 and alpha-smooth muscle actin expression by interleukin-1 alpha and tumour necrosis factor alpha in hepatic stellate cells. Cytotechnology.

[41] Tang R.F., Wang S.X., Zhang F.R., Peng L., Wang S.X., Xiao Y., Zhang M. (2005). Interleukin-1alpha, 6 regulate the secretion of vascular endothelial growth factor A, C in pancreatic cancer. Hepatobiliary Pancreat. Dis. Int..

[42] Laurila E.M., Kallioniemi A. (2013). The diverse role of miR-31 in regulating cancer associated phenotypes. Genes Chromosomes Cancer.

[43] Yuan Y., Wang Z., Chen M., Jing Y., Shu W., Xie Z., Li Z., Xu J., He F., Jiao P. (2021). Macrophage-Derived Exosomal miR-31-5p Promotes Oral Squamous Cell Carcinoma Tumourigenesis Through the Large Tumor Suppressor 2-Mediated Hippo Signalling Pathway. J. Biomed. Nanotechnol..

[44] Zhong Z., Dong Z., Yang L., Chen X., Gong Z. (2013). MicroRNA-31-5p modulates cell cycle by targeting human mutL homolog 1 in human cancer cells. Tumour Biol..

[45] Yuan Y., Shi X., Li B., Peng M., Zhu T., Lv G., Liu L., Jin H., Li L., Qin D. (2020). Integrated analysis of key microRNAs /TFs /mRNAs/ in HPV-positive cervical cancer based on microRNA sequencing and bioinformatics analysis. Pathol. Res. Pract..

[46] Carrera M., Bitu C.C., de Oliveira C.E., Cervigne N.K., Graner E., Manninen A., Salo T., Coletta R.D. (2015). HOXA10 controls proliferation, migration and invasion in oral squamous cell carcinoma. Int. J. Clin. Exp. Pathol..

[47] Wei R., Rodrìguez R.A., Mullor M.D.M.R., Tan Z., Gui Y., Hu J., Zhu T., Huang X., Zhu Y., Xu J. (2020). Analyzing the prognostic value of DKK1 expression in human cancers based on bioinformatics. Ann. Transl. Med..

[48] Vijayakumar G., Narwal A., Kamboj M., Sen R. (2020). Association of SOX2, OCT4 and WNT5A Expression in Oral Epithelial Dysplasia and Oral Squamous Cell Carcinoma: An Immunohistochemical Study. Head Neck Pathol..

[49] Torbrand C., Wigertz A., Drevin L., Folkvaljon Y., Lambe M., Hakansson U., Kirrander P. (2017). Socioeconomic factors and penile cancer risk and mortality; a population-based study. BJU Int..

[50] Grivennikov S.I., Greten F.R., Karin M. (2010). Immunity, inflammation, and cancer. Cell.

[51] Hanahan D., Weinberg R.A. (2011). Hallmarks of cancer: The next generation. Cell.

