# Supplementary material for: Disruption of miRNA-mRNA Networks Defines Novel Molecular Signatures for Penile Carcinogenesis
Source: Cancers (Basel). 2021 Sep 23;13(19):4745. doi: 10.3390/cancers13194745 (PMC8507530; doi:10.3390/cancers13194745)
Supplement: Supplementary file 1 [file cancers-13-04745-s001.zip › cancers-1384996-supplementary.pdf]

Supplementary Material

Table S1. Taqman® Advanced miRNA Assays of the differentially expressed miRNAs (DEmiRs) selected for validation by qRT-PCR.

Type	miRNA	TaqMan® Advanced miRNA Assays
DEmiRs selected for validation	miR-432-5p	478101_mir
	miR-487b-3p	477835_mir
	miR-30a-5p	479448_mir
	miR-149-5p	477917_mir
	miR-145-5p	477916_mir
	miR-200a-5p	478752_mir
	miR-224-5p	477986_mir
	miR-31-3p	478012_mir
	miR-31-5p	478015_mir
Endogenous controls	miR-103a-3p	478253_mir
	miR-423-5p	478090_mir

DEmiR: differentially expressed miRNA; qRT-PCR: quantitative Reverse Transcription Polymerase Chain Reaction.

Table S2. Complete list of the genes included in the panel (83 genes of interest and seven endogenous controls) used to evaluate transcript expression levels in all tumoral (N=24) and non-neoplastic (N=24) tissues from PeC patients.

Gene symbol	Gene Name	Classification
ABCB1	*ATP binding cassette subfamily B member 1*	Gene of interest
ACTB	*Actin Beta*	Endogenous control
AKT1	*AKT serine/threonine kinase 1*	Gene of interest
ALDH1A1	*aldehyde dehydrogenase 1 family member A1*	Gene of interest
ATM	*ATM serine/threonine kinase*	Gene of interest
B2M	*Beta-2-Microglobulin*	Endogenous control
BAX	*BCL2 associated X, apoptosis regulator*	Gene of interest
BBC3	*BCL2 binding component 3*	Gene of interest
BCL2	*BCL2, apoptosis regulator*	Gene of interest
CCND1	*cyclin D1*	Gene of interest
CD274	*CD274 molecule*	Gene of interest
CD44	*CD44 molecule (Indian blood group)*	Gene of interest
CDH1	*cadherin 1*	Gene of interest
CDH2	*cadherin 2*	Gene of interest
CDKN1A	*cyclin dependent kinase inhibitor 1A*	Gene of interest
CDKN2A	*cyclin dependent kinase inhibitor 2A*	Gene of interest
CEBPB	*CCAAT/enhancer binding protein beta*	Gene of interest
CXCR4	*C-X-C motif chemokine receptor 4*	Gene of interest
DKK1	*dickkopf WNT signaling pathway inhibitor 1*	Gene of interest
DNMT1	*DNA methyltransferase 1*	Gene of interest
EGFR	*epidermal growth factor receptor*	Gene of interest
EGR1	*early growth response 1*	Gene of interest
ENTPD5	*ectonucleoside triphosphate diphosphohydrolase 5*	Gene of interest
ESRP1	*epithelial splicing regulatory protein 1*	Gene of interest
FGF2	*fibroblast growth factor 2*	Gene of interest
FOS	*Fos proto-oncogene, AP-1 transcription factor subunit*	Gene of interest
GADD45A	*growth arrest and DNA damage inducible alpha*	Gene of interest
GAPDH	*Glyceraldehyde-3-Phosphate Dehydrogenase*	Endogenous control
GUSB	*Glucuronidase Beta*	Endogenous control
HIF1A	*hypoxia inducible factor 1 alpha subunit*	Gene of interest
HOXA10	*homeobox A10*	Gene of interest
HOXA9	*homeobox A9*	Gene of interest
HPRT1	*Hypoxanthine Phosphoribosyltransferase 1*	Endogenous control
IGF1R	*insulin like growth factor 1 receptor*	Gene of interest
IL1A	*interleukin 1 alpha*	Gene of interest
IL1B	*interleukin 1 beta*	Gene of interest

IL6	*interleukin 6*	Gene of interest
JUN	*Jun proto-oncogene, AP-1 transcription factor subunit*	Gene of interest
KAT6A	*lysine acetyltransferase 6A*	Gene of interest
KLF4	*Kruppel like factor 4*	Gene of interest
KMT2A	*lysine methyltransferase 2A*	Gene of interest
KMT2D	*lysine methyltransferase 2D*	Gene of interest
KRAS	*KRAS proto-oncogene, GTPase*	Gene of interest
LATS2	*large tumor suppressor kinase 2*	Gene of interest
LIN28A	*lin-28 homolog A*	Gene of interest
MCM2	*minichromosome maintenance complex component 2*	Gene of interest
MDM2	*MDM2 proto-oncogene*	Gene of interest
MLH1	*mutL homolog 1*	Gene of interest
MMP1	*matrix metallopeptidase 1*	Gene of interest
MMP12	*matrix metallopeptidase 12*	Gene of interest
MMP9	*matrix metallopeptidase 9*	Gene of interest
MYC	*v-myc avian myelocytomatosis viral oncogene homolog*	Gene of interest
MYD88	*myeloid differentiation primary response 88*	Gene of interest
NANOG	*Nanog homeobox*	Gene of interest
NES	*nestin*	Gene of interest
NOTCH1	*notch 1*	Gene of interest
NOTCH4	*notch 4*	Gene of interest
NRP1	*neuropilin 1*	Gene of interest
PEBP1	*phosphatidylethanolamine binding protein 1*	Gene of interest
PITX2	*paired like homeodomain 2*	Gene of interest
PMAIP1	*phorbol-12-myristate-13-acetate-induced protein 1*	Gene of interest
PPARGC1A	*PPARG coactivator 1 alpha*	Gene of interest
PTEN	*phosphatase and tensin homolog*	Gene of interest
Rb1	*RB transcriptional corepressor 1*	Gene of interest
RECK	*reversion inducing cysteine rich protein with kazal motifs*	Gene of interest
RHOA	*ras homolog family member A*	Gene of interest
RIPK3	*receptor interacting serine/threonine kinase 3*	Gene of interest
RPLP0	*Ribosomal Protein Lateral Stalk Subunit P0*	Endogenous control
SAV1	*salvador family WW domain containing protein 1*	Gene of interest
SESN2	*sestrin 2*	Gene of interest
SFN	*stratifin*	Gene of interest
SNAI1	*snail family transcriptional repressor 1*	Gene of interest
SNAI2	*snail family transcriptional repressor 2*	Gene of interest
SOX2	*SRY-box 2*	Gene of interest
SRC	*SRC proto-oncogene, non-receptor tyrosine kinase*	Gene of interest

STAT3	*signal transducer and activator of transcription 3*	Gene of interest
STK4	*serine/threonine kinase 4*	Gene of interest
TCF7L2	*transcription factor 7like 2*	Gene of interest
TFRC	*Transferrin Receptor*	Endogenous control
TGFB1	*transforming growth factor beta 1*	Gene of interest
TIGAR	*TP53 induced glycolysis regulatory phosphatase*	Gene of interest
TLR4	*toll like receptor 4*	Gene of interest
TNFRSF1A	*TNF receptor superfamily member 1A*	Gene of interest
TP53	*tumor protein p53*	Gene of interest
TP53I3	*tumor protein p53 inducible protein 3*	Gene of interest
TP63	*tumor protein p63*	Gene of interest
TWIST1	*twist family bHLH transcription factor 1*	Gene of interest
VEGFA	*vascular endothelial growth factor A*	Gene of interest
WNT5a	*Wnt family member 5A*	Gene of interest
ZEB1	*zinc finger E-box binding homeobox 1*	Gene of interest

Table S3. Total number of PeC patients included in the study and reasons for exclusion of 17 patients.

**Patients of the study**	**N**
Number of PeC patients at ICESP	41
Reasons for exclusion of patients	17
Patient refused to sign informed consent	2
Patient refused penectomy	1
Death before treatment	3
Advanced disease	2
Previous treatment for PeC	1
HIV or HCV infection	2
Penectomy performed in another Hospital	2
Insufficient material	3
Lost to follow-up	1
Total number of included patients	24

N: number of patients; ICESP: Instituto do Câncer do Estado de São Paulo; PeC: penile carcinoma; HIV: human immunodeficiency virus; HCV: hepatitis C virus.

Table S4. List of 69 DEmiRs identified by microarray analysis in the comparison between TT (N=11) and NNT (N=11) tissues.

Reg	DEmiR	FC	p	FDR
Downregulation	miR-432-5p	-13.44	<0.0001	0.0048
	miR-487b-3p	-8.52	0.0001	0.0079
	miR-139-5p	-7.37	<0.0001	0.0048
	miR-497-5p	-7.28	<0.0001	0.0067
	miR-509-3p	-6.95	0.0003	0.0214
	miR-30a-3p	-6.81	0.0005	0.0289
	miR-127-3p	-6.46	<0.0001	0.0048
	miR-139-3p	-6.38	<0.0001	0.0048
	miR-382-5p	-5.60	0.0006	0.0327
	miR-145-5p	-5.42	0.0001	0.0088
	miR-379-5p	-5.39	<0.0001	0.0067
	miR-125b-2-3p	-5.35	0.0001	0.0096
	miR-196a-5p	-5.10	0.0005	0.0308
	miR-195-5p	-4.93	<0.0001	0.0064
	miR-433-3p	-4.92	0.0001	0.0079
	miR-134-5p	-4.89	0.0001	0.0095
	miR-143-3p	-4.88	0.0001	0.0107
	miR-125b-5p	-4.83	<0.0001	0.0067
	miR-30a-5p	-4.67	<0.0001	0.0067
	miR-409-3p	-4.67	0.0002	0.0141
	miR-99a-5p	-4.46	0.0002	0.0141
	miR-100-5p	-4.46	0.0002	0.0162
	miR-485-5p	-4.31	<0.0001	0.0067
	miR-214-3p	-3.86	<0.0001	0.0057
	miR-149-5p	-3.46	0.0006	0.0308
	miR-10b-5p	-3.31	0.0007	0.0368
	miR-140-3p	-3.17	<0.0001	0.0048
	miR-494-3p	-2.95	0.0004	0.0247
	miR-199a-5p	-2.94	0.0002	0.0162
	miR-199a-3p	-2.92	0.0003	0.0221
	miR-199b-3p	-2.92	0.0003	0.0221
	miR-574-3p	-2.75	<0.0001	0.0048
	miR-126-3p	-2.40	0.0002	0.0125
	let-7e-5p	-2.38	0.0001	0.0111
	miR-378a-3p	-2.33	0.0001	0.0090
	miR-26a-5p	-2.31	0.0001	0.0111
	miR-378c	-2.27	0.0013	0.0492
	let-7c-5p	-2.21	0.0008	0.0405
	miR-500a-3p	-2.19	<0.0001	0.0056
	miR-361-5p	-2.07	0.0010	0.0451
	miR-34a-5p	-2.03	0.0004	0.0238
Upregulation	miR-936	2.01	<0.0001	0.0048
	miR-1184	2.15	<0.0001	0.0056
	miR-4721	2.16	0.0010	0.0458
	miR-8073	2.18	0.0010	0.0451
	miR-4322	2.23	0.0005	0.0298
	miR-6813-5p	2.26	0.0003	0.0207
	miR-4443	2.48	0.0010	0.0451
	miR-18a-5p	2.61	0.0006	0.0322
	miR-6768-5p	2.67	0.0012	0.0492
	miR-4640-5p	2.82	0.0012	0.0490
	miR-4539	2.90	0.0001	0.0107
	miR-200a-5p	2.93	<0.0001	0.0048
	miR-21-3p	2.97	0.0001	0.0075
	miR-3687	2.98	<0.0001	0.0058
	miR-7162-3p	3.22	0.0001	0.0118
	miR-25-5p	3.30	<0.0001	0.0075
	miR-1910-5p	3.36	0.0001	0.0088
	miR-8064	3.68	0.0007	0.0368
	miR-4793-3p	4.01	0.0011	0.0490
	miR-183-5p	4.04	0.0012	0.0492
	miR-5195-3p	4.73	0.0001	0.0124
	miR-365b-5p	4.77	0.0011	0.0467
	miR-4485	5.75	<0.0001	0.0033
	miR-224-5p	6.56	0.0001	0.0083
	miR-187-3p	8.19	<0.0001	0.0048
	miR-4417	10.66	0.0001	0.0083
	miR-31-3p	12.37	<0.0001	0.0002
	miR-31-5p	38.24	<0.0001	0.0048

DEmiR: differentially expressed miRNA; TT: tumor tissue; NNT: non-neoplastic tissue; Reg: regulation; FC: fold change; FDR: false discovery rate

Table S5. Top ten enriched pathways of the experimentally validated target genes (weak or strong functional evidence) of the DEmiRs detected in the comparison between tumoral (N=24) and non-neoplastic (N=24) tissues according to miRTarBase 8.0 database.

Regulation	miRNA	REACTOME ID	Description	p-value	FDR	Genes	Count
Downregulated miRNAs	hsa-miR-145-5p	R-HSA-5663202	Diseases of signal transduction	2,58E-11	1,45E-08	*CDKN1A, STAT1, IRS1, EGFR, MYC, IRS2, JAG1, NRAS, MDM2, ADAM17, HDAC2, KREMEN1, APH1A, BRAF, ACTB, SMAD3, TGFBR2, SMAD4, MAP3K11, ERBB4, ZFYVE9, CD28, HDAC11, FZD6, AGTRAP, HBEGF, SMAD2*	27
	hsa-miR-145-5p	R-HSA-2262752	Cellular responses to stress	1,34E-04	3,95E-03	*CDKN1A, IFNB1, CDK4, VEGFA, MDM2, MAP2K6, EPAS1, ETS1, MAP2K4, SP1, CDK6, HMGA2, E2F3, RPS6KA3, NUP43, RPA1, HIF1A, HIST1H2BF*	18
	hsa-miR-145-5p	R-HSA-2559583	Cellular Senescence	1,16E-05	5,93E-04	*CDKN1A, IFNB1, CDK4, MDM2, MAP2K6, ETS1, MAP2K4, SP1, CDK6, HMGA2, E2F3, RPS6KA3, HIST1H2BF*	13
	hsa-miR-145-5p	R-HSA-9006936	Signaling by TGF-beta family members	4,25E-06	3,89E-04	*MYC, SERPINE1, F11R, SMAD3, SMAD5, TGFBR2, SMAD4, SP1, ZFYVE9, SMAD2*	10
	hsa-miR-145-5p	R-HSA-6785807	Interleukin-4 and 13 signaling	7,12E-06	5,00E-04	*SOX2, MUC1, CDKN1A, STAT1, FSCN1, MYC, VEGFA, MMP1, NANOG, HIF1A*	10
	hsa-miR-145-5p	R-HSA-8878166	Transcriptional regulation by RUNX2	1,95E-05	9,14E-04	*CDKN1A, STAT1, YES1, CBFB, PPM1D, CDK4, ESR1, SOX9, SMAD4, SP7*	10
	hsa-miR-145-5p	R-HSA-170834	Signaling by TGF-beta Receptor Complex	1,91E-06	2,14E-04	*MYC, SERPINE1, F11R, SMAD3, TGFBR2, SMAD4, SP1, ZFYVE9, SMAD2*	9
	hsa-miR-145-5p	R-HSA-8848021	Signaling by PTK6	1,75E-06	2,14E-04	*EGFR, CDK4, NRAS, EPAS1, ERBB4, HBEGF, HIF1A, PXN*	8
	hsa-miR-145-5p	R-HSA-9006927	Signaling by Non-Receptor Tyrosine Kinases	1,75E-06	2,14E-04	*EGFR, CDK4, NRAS, EPAS1, ERBB4, HBEGF, HIF1A, PXN*	8
	hsa-miR-145-5p	R-HSA-2219528	PI3K/AKT Signaling in Cancer	8,74E-05	3,03E-03	*CDKN1A, IRS1, EGFR, IRS2, MDM2, ERBB4, CD28, HBEGF*	8

Continuation of Table S5

Regulation	miRNA	REACTOME ID	Description	p-value	FDR	Genes	Count
Upregulated miRNAs	hsa-miR-31-5p	R-HSA-194315	Signaling by Rho GTPases	6,61E-05	6,85E-03	*RHOA, ARPC5, TIAM1, WASF3, HIST1H2BC, HIST1H2BK, FLNA, RANGAP1, HIST1H2BJ, SRC, YWHAE, RHOBTB1, NF2, AR, SYDE2, GNA13, SFN*	17
	hsa-miR-31-5p	R-HSA-5663205	Infectious disease	3,82E-05	6,60E-03	*MET, RPS7, NUP188, RPS4Y1, RPL37A, RANGAP1, SRC, ARF1, RPL35A, GTF2E1, CCNT1, RPL27A, RPL12, XRCC5, AP2B1, NPM1*	16
	hsa-miR-31-5p	R-HSA-195258	RHO GTPase Effectors	2,55E-04	9,44E-03	*RHOA, ARPC5, WASF3, HIST1H2BC, HIST1H2BK, FLNA, RANGAP1, HIST1H2BJ, SRC, YWHAE, NF2, AR, SFN*	13
	hsa-miR-224-5p	R-HSA-5683057	MAPK family signaling cascades	5,45E-05	7,66E-03	*CDC42, PDGFRB, PEBP1, KRAS, TNRC6A, PAK2, PSMD3, NCOA3, XPO1, MAP2K2, RAC1*	11
	hsa-miR-31-5p	R-HSA-927802	Nonsense-Mediated Decay (NMD)	1,32E-04	9,44E-03	*PPP2R2A, RPS7, RPS4Y1, RPL37A, SMG1, RPL35A, RPL27A, RPL12*	8
	hsa-miR-31-5p	R-HSA-975957	Nonsense Mediated Decay (NMD) enhanced by the Exon Junction Complex (EJC)	1,32E-04	9,44E-03	*PPP2R2A, RPS7, RPS4Y1, RPL37A, SMG1, RPL35A, RPL27A, RPL12*	8
	hsa-miR-31-5p	R-HSA-8878166	Transcriptional regulation by RUNX2	1,77E-04	9,44E-03	*ITGA5, SATB2, CDK1, GLI2, SRC, SP7, AR, SMAD4*	8
	hsa-miR-224-5p	R-HSA-5687128	MAPK6/MAPK4 signaling	1,48E-05	4,17E-03	*CDC42, TNRC6A, PAK2, PSMD3, NCOA3, XPO1, RAC1*	7
	hsa-miR-31-5p	R-HSA-2682334	EPH-Ephrin signaling	1,91E-04	9,44E-03	*RHOA, ARPC5, TIAM1, RASA1, SRC, AP2B1, EFNB1*	7
	hsa-miR-31-5p	R-HSA-69473	G2/M DNA damage checkpoint	2,33E-04	9,44E-03	*CDK1, HIST1H2BC, HIST1H2BK, RPA1, HIST1H2BJ, YWHAE, SFN*	7

FDR: false discovery rate; Pathways are provided by Reactome database for humans and are described separately for each type of regulation (downregulated or upregulated miRNAs).

Table S6. List of Gene Ontology (GO) enriched pathways of differentially expressed genes detected by Biomark HD Fluidigm platform in the comparison between tumoral (N=24) and non-neoplastic tissues (N=24).

Description	setSize	enrichment Score	NES	pvalue	p.adjust	qvalues	rank	Leading edge	Core enrichment
GO RESPONSE TO GROWTH FACTOR	13	-0,74	-2,02	0,00	0,14	0,14	9	tags=54%, list=24%, signal=63%	*ZEB1, KLF4, EGR1, TWIST1, FOS, FGF2, PPARGC1A*
GO MUSCLE TISSUE DEVELOPMENT	10	-0,67	-1,76	0,01	0,53	0,52	7	tags=50%, list=19%, signal=56%	*EGR1, TWIST1, FOS, FGF2, PPARGC1A*
GO CELLULAR RESPONSE TO ENDOGENOUS STIMULUS	13	-0,63	-1,74	0,01	0,53	0,52	12	tags=54%, list=32%, signal=56%	*TLR4, ZEB1, KLF4, EGR1, FOS, FGF2, PPARGC1A*
GO DNA METABOLIC PROCESS	10	-0,64	-1,68	0,02	0,53	0,52	8	tags=50%, list=22%, signal=54%	*KLF4, TWIST1, FOS, FGF2, PPARGC1A*
GO PROTEOLYSIS	12	0,53	1,61	0,02	0,53	0,52	10	tags=58%, list=27%, signal=63%	*MMP1, MMP12, SFN, VEGFA, MDM2, STAT3, RHOA*
GO CIS REGULATORY REGION SEQUENCE SPECIFIC DNA BINDING	11	-0,60	-1,62	0,03	0,76	0,76	16	tags=73%, list=43%, signal=59%	*TCF7L2, NANOG, PITX2, ZEB1, KLF4, EGR1, TWIST1, FOS*
GO TRANSCRIPTION REGULATOR ACTIVITY	18	-0,54	-1,57	0,04	0,78	0,77	17	tags=61%, list=46%, signal=64%	*KMT2A, TCF7L2, NANOG, PITX2, ZEB1, KLF4, EGR1, TWIST1, FOS, FGF2, PPARGC1A*
GO DNA BINDING TRANSCRIPTION FACTOR ACTIVITY	13	-0,57	-1,56	0,05	0,78	0,77	17	tags=69%, list=46%, signal=58%	*KMT2A, TCF7L2, NANOG, PITX2, ZEB1, KLF4, EGR1, TWIST1, FOS*
GO RESPONSE TO ENDOGENOUS STIMULUS	15	-0,56	-1,59	0,05	0,78	0,77	14	tags=53%, list=38%, signal=56%	*BCL2, TLR4, ZEB1, KLF4, EGR1, FOS, FGF2, PPARGC1A*
GO CHROMATIN	14	-0,55	-1,53	0,06	0,78	0,77	16	tags=64%, list=43%, signal=59%	*TCF7L2, NANOG, PITX2, ZEB1, KLF4, EGR1, TWIST1, FOS, PPARGC1A*
GO TRANSITION METAL ION BINDING	10	0,53	1,56	0,06	0,78	0,77	3	tags=30%, list=8%, signal=38%	*MMP1, IL1A, MMP12*
GO RESPONSE TO ORGANIC CYCLIC COMPOUND	10	-0,56	-1,47	0,07	0,82	0,82	8	tags=40%, list=22%, signal=43%	*KLF4, EGR1, FOS, PPARGC1A*
GO SEQUENCE SPECIFIC DNA BINDING	16	-0,53	-1,51	0,08	0,82	0,82	17	tags=69%, list=46%, signal=65%	*KMT2A, TCF7L2, NANOG, BCL2, PITX2, ZEB1, KLF4, EGR1, TWIST1, FOS, PPARGC1A*
GO NUCLEAR CHROMOSOME	15	-0,53	-1,49	0,08	0,85	0,84	16	tags=60%, list=43%, signal=57%	*TCF7L2, NANOG, PITX2, ZEB1, KLF4, EGR1, TWIST1, FOS, PPARGC1A*
GO POSITIVE REGULATION OF MULTICELLULAR ORGANISMAL PROCESS	18	-0,50	-1,45	0,09	0,85	0,84	9	tags=39%, list=24%, signal=57%	*ZEB1, KLF4, EGR1, TWIST1, FOS, FGF2, PPARGC1A*

Pathways provided by Gene Ontology (GO) database ($p < 0.1$)

Table S7. List of the 69 DEmiRs detected in the comparison of tumoral and non-neoplastic tissues in the microarray analysis with opposite expression levels relative to that of the 37 DEGs described as experimentally validated according to miRTarBase 8.0

DEGs	DEmiRs
ABCB1	-
ALDH1A1	-
BCL2	**miR-18a-5p, miR-224-5p***
CCND1	**miR-18a-5p, miR-21-3p, miR-183-5p, miR-224-5p*, miR-6768-5p**
EGR1	**miR-183-5p**
FGF2	**miR-936**
FOS	**miR-4640-5p**
HOXA9	**miR-18a-5p**
IL1A	**miR-30a-5p***
KAT6A	-
KLF4	-
KMT2A	**miR-6813-5p**
LATS2	**miR-31-5p*, miR-183-5p**
MCM2	**miR-145-5p***
MDM2	**miR-4793-3p**
MLH1	**miR-31-5p*, miR-4793-3p**
MMP1	**miR-145-5p***
MMP12	**miR-145-5p***
NANOG	**miR-8073**
NRP1	-
PEBP1	**miR-224-5p***
PITX2	-
PPARGC1A	**miR-8064**
PTEN	**miR-18a-5p, miR-21-3p, miR-200a-5p***
RECK	**miR-183-5p**
RHOA	**miR-31-3p*, miR-31-5p*, miR-4640-5p, miR-4721**
RIPK3	-
SAV1	-
SFN	-
STAT3	**miR-21-3p, miR-365b-5p**
STK4	**miR-18a-5p, miR-4793-3p**
TCF7L2	-
TLR4	-
TP53	**miR-18a-5p**
TWIST1	-
VEGFA	**miR-126-3p, miR-134-5p, miR-145-5p*, miR-195-5p, miR-199a-3p, miR-199a-5p, miR-361-5p, miR-378a-3p, miR-497-5p**
ZEB1	**miR-183-5p**

DEmiR: differentially expressed miRNA; DEG: differentially expressed gene; *DEmiRs detected in microarray analysis and validated by qRT-PCR

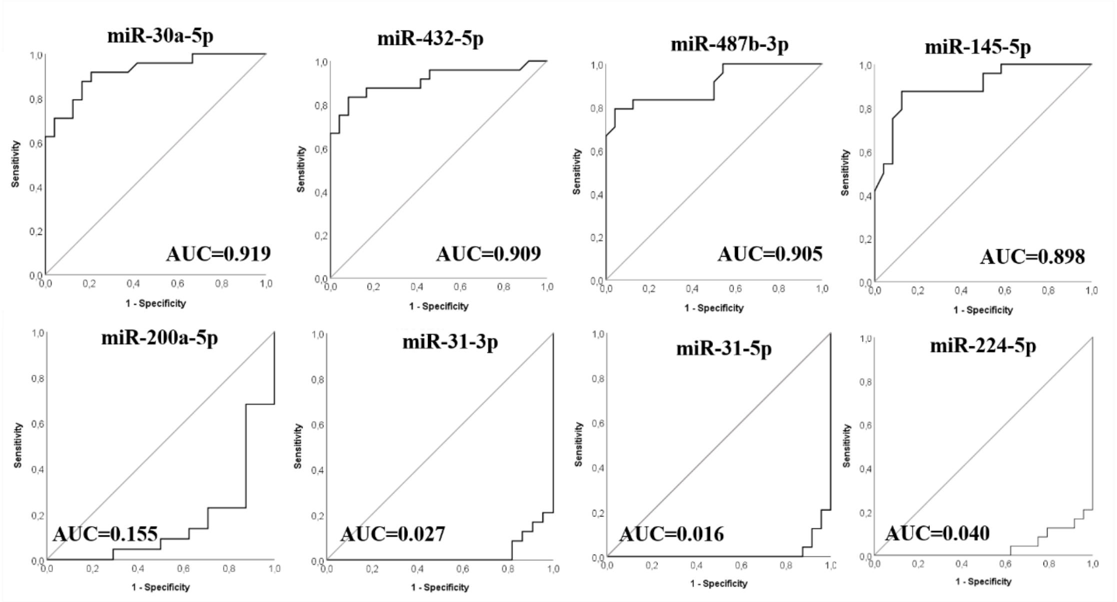

Figure S1. ROC curves of the eight differentially expressed miRNAs (DEmiRs) validated by qRT-PCR. All downregulated DEmiRs (miR-30a-5p, miR-432-5p, miR-487b-3p and miR-145-5p) demonstrated to have good accuracy to discriminate tumoral from non-neoplastic tissues in PeC patients; ROC: receiver operating characteristic; AUC: area under the curve.

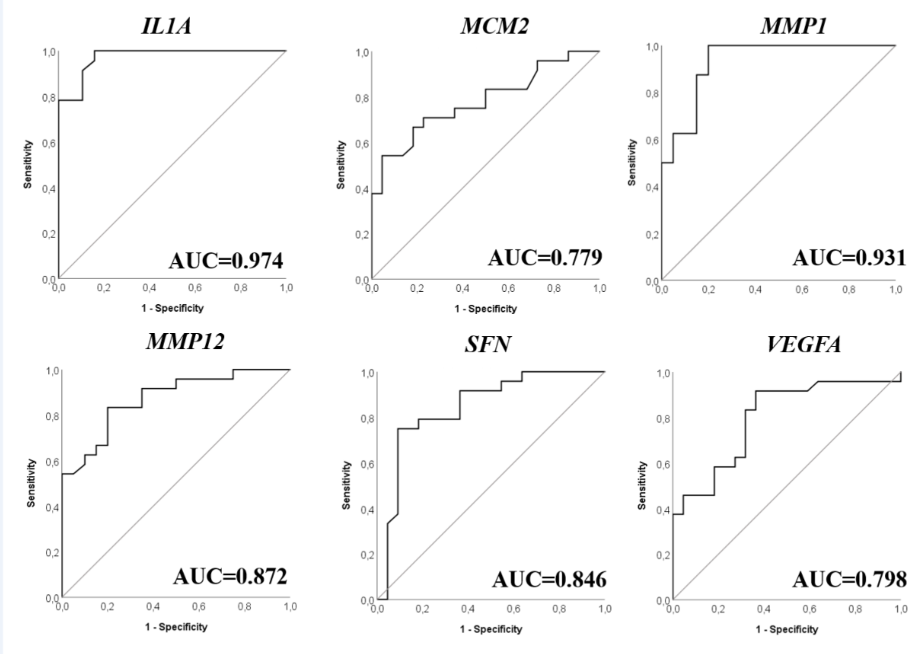

Figure S2. ROC curves of differentially expressed genes (DEGs) that demonstrated to have good accuracy to discriminate tumoral from non-neoplastic tissues (*IL1A*, *MCM2*, *MMP1*, *MMP12*, *SFN* and *VEGFA*) in PeC patients; ROC: receiver operating characteristic; AUC: area under the curve.

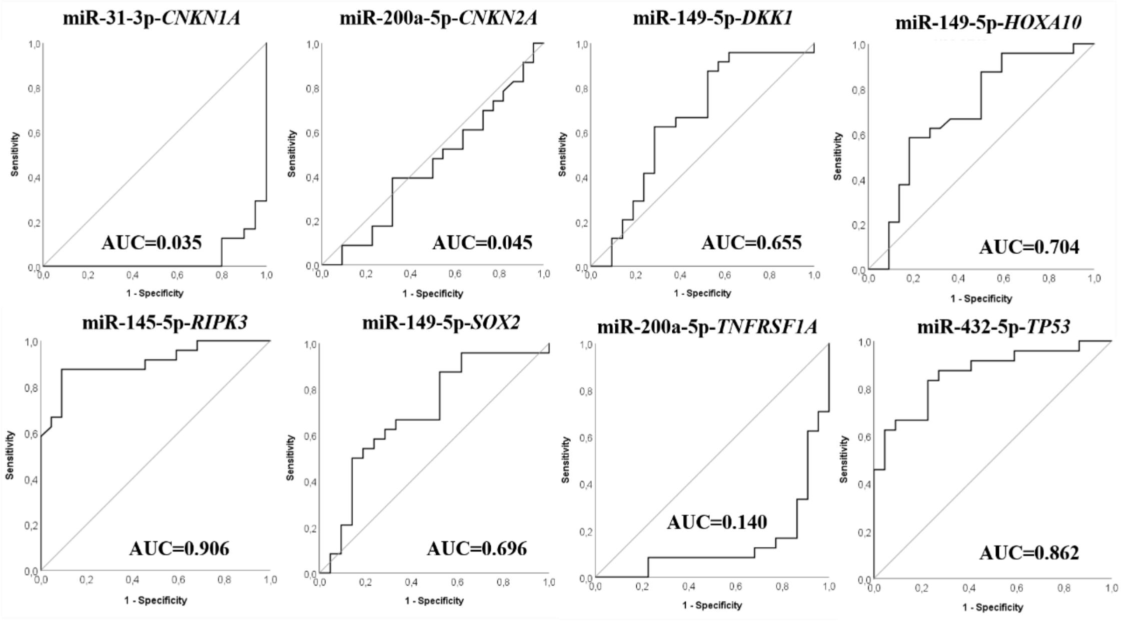

Figure S3. Eight miRNA-mRNA pairs disrupted in the penile carcinogenesis and their respective ROC curves to analyze accuracy in discriminating tumoral from non-neoplastic tissues; ROC: receiver operating characteristic; AUC: area under the curve.
